# Detecting disease-associated genomic outcomes using constrained mixture of Bayesian hierarchical models for paired data

**DOI:** 10.1371/journal.pone.0174602

**Published:** 2017-03-30

**Authors:** Yunfeng Li, Jarrett Morrow, Benjamin Raby, Kelan Tantisira, Scott T. Weiss, Wei Huang, Weiliang Qiu

**Affiliations:** 1 School of Mathematical Sciences, Zhejiang University, HongZhou, Zhejiang, China; 2 Channing Division of Network Medicine, Brigham and Women’s Hospital/Harvard Medical School, Boston, MA, United States of America; National Taiwan University, TAIWAN

## Abstract

Detecting disease-associated genomic outcomes is one of the key steps in precision medicine research. Cutting-edge high-throughput technologies enable researchers to unbiasedly test if genomic outcomes are associated with disease of interest. However, these technologies also include the challenges associated with the analysis of genome-wide data. Two big challenges are (1) how to reduce the effects of technical noise; and (2) how to handle the curse of dimensionality (i.e., number of variables are way larger than the number of samples). To tackle these challenges, we propose a constrained mixture of Bayesian hierarchical models (MBHM) for detecting disease-associated genomic outcomes for data obtained from paired/matched designs. Paired/matched designs can effectively reduce effects of confounding factors. MBHM does not involve multiple testing, hence does not have the problem of the curse of dimensionality. It also could borrow information across genes so that it can be used for whole genome data with small sample sizes.

## Introduction

We propose to develop Bayesian statistical models to identify genomic outcomes associated with complex human diseases, such as cancer and other chronic diseases, that are causing significant burden to patients, families, societies and countries. Identifying disease-associated genomic outcomes could not only help discover the underlying molecular mechanisms of complex human diseases, but also help explain the inter-individual variation of response to drug treatments. It is the first step toward precision medicine that takes into account individual variability in genes, environment, and lifestyle for each person in delivering treatment and prevention measures. Messenger RNA (mRNA) could reflect the effects of both genetic and environmental factors on complex human diseases. By comparing the mRNA abundance between diseased subjects and normal subjects, researchers can identify potential disease-associated genes.

Cutting-edge DNA microarray technology has been developed to simultaneously measure the intensities of mRNAs for tens of thousands of genes in the human genome. This whole-genome approach, unlike the candidate gene approach, could unbiasedly evaluate the associations of tens of thousands of genes to the disease of interest.

When analyzing whole-genome gene expression data, researchers face two big challenges: the effects of noise (e.g., batch effect) in the microarray data and the curse of dimensionality (i.e., the number of predictors (gene probes) is much larger than the number of observations (samples)).

The noise (e.g., batch effect) could either mask the true gene differential expression or create false detection of gene differential expression. Several effective noise-reduction methods, such as quantile normalization [1] and surrogate variable analysis [2], have been proposed for gene microarray data analysis.

Paired/matched designs can also reduce the effects of noise. Paired designs are common in intervention studies, such as clinical trials. Matched designs are common in observational studies, such as matched case-control studies. Both are designed to reduce the effects of potential inter-individual variations by providing a homogeneous environment (i.e., block) for comparing measurements under different conditions. Paired /matched designs are commonly used in gene microarray studies.

The most common method to analyze gene microarray data from paired/matched designs is to perform paired t-test or a moderated paired t-test for one gene probe at a time, then adjust the p-values to control for multiple testing. For example, the R packages *limma* and *samr* from the Bioconductor project [3] utilize this approach (c.f. [4] and [5]). Another approach is regularized regression, such as LASSO (c.f. [6] and [7]). Both approaches aim to reduce the effects of the curse of high dimensionality.

Researchers also used probe clustering, based on mixtures of Bayesian hierarchical models (*MBHMs*) ([8], [9], and [10]), to identify differentially expressed (DE) gene probes. Probe clustering based on *MBHMs* treats gene probes as “samples” and arrays as “variables”. Hence, the number of “samples” (i.e., gene probes) would be much greater than the number of “variables” (i.e., arrays). Therefore, probe clustering based on *MBHMs* does not have the curse-of-dimensionality problem. In addition, unlike probe-specific tests that have several parameters per probe, probe clustering based on *MBHMs* has only a few parameters per cluster and could borrow information across probes to estimate model parameters. Hence it could produce more accurate estimates of model parameters and could work well for datasets with small sample sizes. This property is particularly useful for genomic data that usually have small sample sizes due to high cost of obtaining genome-wide data. Probe clustering based on *MBHMs* is a special type of model-based clustering that has a known number of clusters (2 or 3 clusters) and imposes special restrictions on the structure of mean vectors and covariance matrices for each cluster [11]. By utilizing this additional information about the number of clusters and structures of mean vectors and covariance matrices, probe clustering based on *MBHMs* could have much better performance than probe-clustering algorithms without using this information [11].

Although paired/matched designs are common and very useful in gene microarray studies, to the best of our knowledge, there is no probe clustering method based on *MBHMs* previously developed for analyzing data from these two designs. For example, the probe clustering algorithms based on *MBHMs* proposed in the literature ([8], [9], and [10]) require that samples are independent (c.f. Section A in S1 File). Hence, they could not analyze data in which samples are dependent within a pair. In this paper, we propose a novel *MBHM* method to perform probe clustering for genomic data collected from paired/matched design. Specifically, we propose a constrained *MBHM*, called *eLNNpaired*, to identify disease-associated genetic outcomes measured from paired/matched designs.

## Materials and methods

### eLNNpaired model

We denote *x*_*gl*_ and *y*_*gl*_ as the expression levels of the *g*-th gene probe for the *l*-th sample under two different conditions (e.g., controls and cases). The eLNN model [10] characterizes the hierarchical distributions of *x*_*gl*_ and *y*_*gl*_ and assumes that *x*_*gl*_ and *y*_*gl*_ are independent for a given gene probe *g*. For data from a paired/matched design, samples within a pair are dependent. Hence, the eLNN model could not be used for data from a paired/matched design. To overcome this limitation, we propose to characterize the distribution of the within-pair difference. We denote *d*_*gl*_ as the difference between log_2_ *y*_*gl*_ and log_2_ *x*_*gl*_, that is *d*_*gl*_ = log_2_ *y*_*gl*_ − log_2_ *x*_*gl*_. We assume that the log_2_ difference *d*_*gl*_ is normally distributed. We also assume that each gene probe could be classified into one of 3 clusters: (1) probes over-expressed (OE) in cases; (2) probes under-expressed (UE) in cases; and (3) probes non-differentially expressed (NE) between cases and controls. We further assume a Bayesian hierarchical model for each of the three gene-probe clusters.

For a given probe in cluster 1 (cluster of OE gene probes), we expect that its population mean of log2 difference would be positive. To get a closed-form marginal distribution, we use conjugate prior distributions and assume the following Bayesian hierarchical model:

$$\begin{array}{c} \begin{array}{c} d_{gl}\left|\left(\mu_{g},\tau_{g}\right)∼N\left(\mu_{g},\tau_{g}^{-1}\right),\;\mu_{g}\right|\tau_{g}∼N\left(\mu_{1},k_{1}\tau_{g}^{-1}\right),\;\tau_{g}∼\Gamma \left(\alpha_{1},\beta_{1}\right), \end{array} \end{array}$$ (1)

where *μ*_1_ > 0, *k*_1_ > 0, *α*_1_ > 0, and *β*_1_ > 0.

For a given probe in cluster 2 (cluster of UE gene probes), we expect that its population mean of log2 difference would be negative. Similar to probes to cluster 1, we assume the following Bayesian hierarchical model:

$$\begin{array}{c} \begin{array}{c} d_{gl}\left|\left(\mu_{g},\tau_{g}\right)∼N\left(\mu_{g},\tau_{g}^{-1}\right),\;\mu_{g}\right|\tau_{g}∼N\left(\mu_{2},k_{2}\tau_{g}^{-1}\right),\;\tau_{g}∼\Gamma \left(\alpha_{2},\beta_{2}\right), \end{array} \end{array}$$ (2)

where *μ*_2_ < 0, *k*_2_ > 0, *α*_2_ > 0, and *β*_2_ > 0.

For a given probe in cluster 3 (cluster of NE gene probes), we expect that its population mean *μ*_*g*_ of log2 difference would be exactly zero. Hence, we assume the following Bayesian hierarchical model:

$$\begin{array}{c} \begin{array}{c} d_{gl}|\tau_{g}∼N\left(0,\tau_{g}^{-1}\right),\;\tau_{g}∼\Gamma \left(\alpha_{3},\beta_{3}\right), \end{array} \end{array}$$ (3)

where *α*_3_ > 0 and *β*_3_ > 0.

The hyper-parameters *α*_*c*_ and *β*_*c*_ are shape and rate parameters for the Gamma distribution, respectively, *c* = 1,2,3. As for *k*_1_ and *k*_2_, intuitively, the variation of *μ*_*g*_ should be smaller than that of *d*_*gl*_. So we have 0 < *k*_1_ < 1 and 0 < *k*_2_ < 1.

### Constraints

Ideally, we should require *μ*_*g*_ > 0 (*μ*_*g*_ < 0) for all probes in cluster 1 (cluster 2). To do so, we can assume a log normal prior distribution for *μ*_*g*_ in cluster 1, for instance. However, a log normal distribution is not a conjugate prior for the mean of a normal distribution. It would increase the computational burden if non-conjugate priors were used. As an alternative, we require the mean of *μ*_*g*_ > 0 (mean of *μ*_*g*_ < 0) for cluster 1 (cluster 2). However, this constraint is not enough. For example, if we generate a random number *μ*_*g*_ from $N(\mu_{1},k_{1}\tau_{g}^{-1})$ with *μ*_1_ = 1, it is possible that *μ*_*g*_ is very close to zero (e.g., *μ*_*g*_ = 0.1) or *μ*_*g*_ < 0 (e.g., *μ*_*g*_ = −0.2). Then it would not be reasonable to claim this probe is from cluster 1, which is the cluster of over-expressed probes. Hence, we would like to avoid this type of mistake as much as possible. To quantify this type of mistake, let’s consider a probe from cluster 3 (cluster of NE probes). We expect that its standardized log2 difference $\left(d_{gl}/\sqrt{\tau_{g}^{-1}}\right)$ would most likely be within the interval [*c*_2_, *c*_1_], where *c*_2_ = Φ^−1^(0.05) and *c*_1_ = Φ^−1^(0.95) are the 5-th and 95-th percentile of the standard normal distributions, respectively. Hence, if a probe is from cluster 1 (cluster of OE probes), we expect that $\mu_{g}/\sqrt{\tau_{g}^{-1}}$ should be >*c*_1_. In other words, we require the probability that makes a mistake that $\mu_{g}/\sqrt{\tau_{g}^{-1}}\leq c_{1}$ is small. Mathematically, we require

$$\begin{array}{c} \text{Pr}\left(\frac{\mu_{g}}{\sqrt{\tau_{g}^{-1}}}\leq c_{1}|\tau_{g}^{-1}\right)<0.05, \end{array}$$

which is equivalent to

$$\begin{array}{c} \tau_{g}>{\left(\frac{c_{1}-\Phi^{-1}\left(0.05\right)\sqrt{k_{1}}}{\mu_{1}}\right)}^{2}. \end{array}$$ (4)

It would be too stringent to require that *τ*_*g*_ for all probes in cluster 1 should satisfy the inequality in Eq (4). So we relax the constraint by requiring that at least the most possible value of *τ*_*g*_ (i.e., mode of *τ*_*g*_) should satisfy the inequality in Eq (4):

$$\begin{array}{c} mode\left(\tau_{g}\right)=\frac{\alpha_{1}-1}{\beta_{1}}>{\left(\frac{c_{1}-\Phi^{-1}\left(0.05\right)\sqrt{k_{1}}}{\mu_{1}}\right)}^{2}, \end{array}$$

which is equivalent to

$$\begin{array}{c} \alpha_{1}>1+\beta_{1}{\left(\frac{c_{1}-\Phi^{-1}\left(0.05\right)\sqrt{k_{1}}}{\mu_{1}}\right)}^{2}, \end{array}$$

where *c*_1_ = Φ^−1^(0.95).

Similarly, for probes in cluster 2 (cluster of UE probes) we require

$$\begin{array}{c} \text{Pr}\left(\frac{\mu_{g}}{\sqrt{\tau_{g}^{-1}}}\geq c_{2}|\tau_{g}^{-1}\right)<0.05 \end{array}$$

and get the following constraint for cluster 2:

$$\begin{array}{c} \alpha_{2}>1+\beta_{2}{\left(\frac{c_{2}-\Phi^{-1}\left(0.95\right)\sqrt{k_{2}}}{\mu_{2}}\right)}^{2}, \end{array}$$

where *c*_2_ = Φ^−1^(0.05).

### Parameter estimation

To make sure the parameters satisfy the constraints in numerical optimization, we re-parameterized the parameters by ***ψ*** = (*δ*_1_, *ξ*_1_, *λ*_1_, *ν*_1_, *δ*_2_, *ξ*_2_, *λ*_2_, *ν*_2_, *λ*_3_, *ν*_3_), where *μ*_1_ = exp(*δ*_1_), *k*_1_ = Φ(*ξ*_1_), *α*_1_ = exp(*λ*_1_), *β*_1_ = exp(*ν*_1_), *μ*_2_ = −exp(*δ*_2_), *k*_2_ = Φ(*ξ*_2_), *α*_2_ = exp(*λ*_2_), *β*_2_ = exp(*ν*_2_), *α*_3_ = exp(*λ*_3_), *β*_3_ = exp(*ν*_3_), and

$$\begin{array}{c} \begin{array}{c} \alpha_{1}=\exp\left(\lambda_{1}\right)+1+\beta_{1}{\left(\frac{c_{1}-\Phi^{-1}\left(0.05\right)\sqrt{k_{1}}}{\mu_{1}}\right)}^{2}, \end{array} \end{array}$$

$$\begin{array}{c} \begin{array}{c} \alpha_{2}=\text{exp}\left(\lambda_{2}\right)+1+\beta_{2}{\left(\frac{c_{2}-\Phi^{-1}\left(0.95\right)\sqrt{k_{2}}}{\mu_{2}}\right)}^{2}, \end{array} \end{array}$$

Φ is the cumulative distribution function of standard normal distribution.

We denote *f*_1_(**d**_*g*_|***ψ***), *f*_2_(**d**_*g*_|***ψ***) and *f*_3_(**d**_*g*_|***ψ***) as the marginal densities of the 3 clusters, respectively. The formulae for these 3 marginal densities are shown in Section B in S1 File. Denote ***π*** = (*π*_1_, *π*_2_, *π*_3_) as the cluster proportions. We impose a symmetric Dirichlet *D*(***b***) prior on *π* with concentration parameters ***b*** = (*b*_1_, *b*_2_, *b*_3_) = (*b*, *b*, *b*) to stabilize the estimate of ***π***. We would like to choose the value for *b* so that the mixture proportions are most likely to be equal (*π*_1_ = *π*_2_ = *π*_3_ = 1/3). Any value *b* > 1 would satisfy this condition since the mode of *D*(***b***) is 1/3, which does not depend on *b*. Following [8], we set *b* = 2. Let **z**_*g*_ = (*z*_*g*1_, *z*_*g*2_, *z*_*g*3_), where *z*_*gc*_ is an indicator variable indicating if gene probe *g* belongs to cluster *c* (*z*_*gc*_ = 1) or not (*z*_*gc*_ = 0), *c* = 1,2,3.

The complete data log-likelihood is:

$$\begin{array}{c} \begin{array}{cc} & l(\pi ,\psi |d,z)\\ = & \sum_{g}\left(z_{g1}\log f_{1}\left(d_{g}|\psi \right)+z_{g2}\log f_{2}\left(d_{g}|\psi \right)+z_{g3}\log f_{3}\left(d_{g}|\psi \right)\right)\\ & +\sum_{g}\left(z_{g1}\log\pi_{1}+z_{g2}\log\pi_{2}+z_{g3}\log\pi_{3}\right)\\ & +\log\left(\frac{\Gamma (\sum_{c=1}^{3}b_{c})}{\prod_{c=1}^{3}\Gamma \left(b_{c}\right)}\right)+\sum_{c=1}^{3}\left(b_{c}-1\right)\log\pi_{c}, \end{array} \end{array}$$ (5)

where **d** = (**d**_1_,…,**d**_*G*_) and **z** = (**z**_1_,…,**z**_*G*_), and *G* is the number of gene probes.

For gene probe *g*, let ${\tilde{z}}_{gc}=\text{Pr}(z_{gc}=1|d_{g},\pi ,\psi )$, *c* = 1, 2, 3. Let ${\tilde{z}}_{g}=\left({\tilde{z}}_{g1},{\tilde{z}}_{g2},{\tilde{z}}_{g3}\right)$. Applying Bayes rule, we get the posterior probability:

$$\begin{array}{c} \begin{array}{cc} {\tilde{z}}_{gc} & =\text{Pr}(z_{gc}=1|d_{g},\pi ,\psi )\\ & =\frac{\text{Pr}\left(d_{g}|g\;\text{is}\;\text{in}\;\text{cluster}\;c\right)\text{Pr}\left(g\;\text{is}\;\text{in}\;\text{cluster}\;c\right)}{\sum_{s}\text{Pr}\left(d_{g}|g\;\text{is}\;\text{in}\;\text{cluster}\;s\right)\text{Pr}\left(g\;\text{is}\;\text{in}\;\text{cluster}\;s\right)}\\ & =\frac{\pi_{c}f_{c}\left(d_{g}|\psi \right)}{\pi_{1}f_{1}\left(d_{g}|\psi \right)+\pi_{2}f_{2}\left(d_{g}|\psi \right)+\pi_{3}f_{3}\left(d_{g}|\psi \right)}, \end{array} \end{array}$$

for *c* = 1, 2, 3.

The EM algorithm is used to estimate parameters ***π*** and ***ψ***. In the E-step, we treat **z**_*g*_ as missing values and integrate out **z**_*g*_ by calculating the expectation of *l*(***π***, ***ψ***|**d**,**z**) w.r.t. **z**_*g*_. In the (*t*+1)-th iteration of the EM algorithm, we have $\text{E}\left[l\left(\pi ,\psi |d,z,\pi^{\left(t\right)},\psi^{\left(t\right)}\right)\right]=l(\pi ,\psi |d,\tilde{z})$, where ***π***^(*t*)^ and ***ψ***^(*t*)^ are estimated in the *t*-th iteration, and $\tilde{z}=({\tilde{z}}_{1},\ldots ,{\tilde{z}}_{G})$. In the M-step, we maximize the expected log likelihood ([*l*(***π***, ***ψ***|**d**,**z**, ***π***^(*t*)^, ***ψ***^(*t*)^)]) over parameters ***π*** and ***ψ***. We repeat these two steps until the difference of the parameters ***π*** and ***ψ*** between two consecutive iterations is small or the number of iterations exceeds the allowed maximum number. Details about the marginal distributions and the EM algorithm are shown in Sections B and C in S1 File. The method to initialize model parameters is shown in Section D in S1 File. The gene probe *g* will be classified to cluster *c* if the posterior probability ${\tilde{z}}_{gc}$ is the largest among ${\tilde{z}}_{g1}$, ${\tilde{z}}_{g2}$, and ${\tilde{z}}_{g3}$.

### Approximated weighted density plot

We intend to plot density functions in one plot with a red line for *π*_1_ *f*_1_(**d**_*g*_|***ψ***), a blue line for *π*_2_ *f*_2_(**d**_*g*_|***ψ***), a black line for *π*_3_ *f*_3_(**d**_*g*_|***ψ***) and brown line for the summation of these three weighted density functions. However, since **d**_*g*_ is a vector of multiple dimensions, it would be very difficult to visualize *π*_1_ *f*_1_(**d**_*g*_|***ψ***), *π*_2_ *f*_2_(**d**_*g*_|***ψ***) and *π*_3_ *f*_3_(**d**_*g*_|*ψ*). To provide a rough plot for these weighted density functions, we set **d**_*g*_ to be one dimension, that is, it only contains information from one sample, to approximate the actual weighted densities.

### GEO datasets

*GSE43292* [12] is from a genome-wide expression study of human carotid atheroma, which contains paired samples for 32 patients. For a given patient, one sample is from the atheroma plaque and the other sample is from distant macroscopically intact tissue. For each of the 64 samples, the expression levels of 33,297 gene probes were measured by using Affymetrix Human Gene 1.0 ST array.

*GSE24742* [13] is from a study investigating the global molecular effects of rituximab in synovial biopsies obtained from 12 anti-TNF resistant rheumatoid arthritis (RA) patients before and after administration of the drug (rituximab). For each of the 24 samples, the expression levels of 54,675 gene probes were measured by Affymetrix Human Genome U133 Plus 2.0 array.

The study associated with *GSE6631* [14] aimed to identify reliable differentially-expressed genes between samples of head and neck squamous cell carcinoma (HNSCC) and normal tissue samples from a study with paired design; paired samples from 44 patients were used to measure expression levels of 12,625 genes using the Affymetrix Human Genome U95 version 2 array.

Table 1 summarizes the numbers of probes, the numbers of sample pairs, and the microarray platforms for the 3 GEO data sets.

**Table 1:** The numbers of probes, the numbers of sample pairs, and platforms for the 3 GEO datasets.

	Number of probes	Number of sample pairs	Platform
GSE43292	33297	32	Affymetrix Human Gene 1.0 ST
GSE24742	54675	12	Affymetrix Human Genome U133 Plus 2.0
GSE6631	12625	22	Affymetrix Human Genome U95 version 2

### QC checking for the GEO data sets

We downloaded datasets from https://www.ncbi.nlm.nih.gov/geo/ and performed quality checking before further analysis. First we checked if there were any missing values, duplicated samples or duplicated subjects. We used *lumiT* in Bioconductor package *lumi* to test if the original dataset had been log_2_ transformed; if not, a log_2_ transformation was performed. We found that *GSE24742* and *GSE6631* were not log_2_ transformed, while *GSE43292* had already been log_2_ transformed. To check the existence of outliers, for each array we calculated its 0-th, 5-th, 25-th, 50-th, 75-th, 95-th, and 100-th percentiles of expression levels and viewed them across all arrays. We also obtained the principal components (PCA) of gene expression data and plotted the first component against the second component. Please refer to Figs A, B and C in S1 File for QC plots of the three datasets. Based on the results, we found that the three datasets had good quality, with no obvious outliers or batch effects.

### Generating simulated datasets

We conducted two sets of simulation studies. In the first set of the simulation studies, log_2_ difference of expression levels within a pair of samples were generated from the *eLNNpaired* model. We used the model parameters estimated from GSE43292 as the true values of the model parameters. That is: *μ*_1_ = 0.441, *k*_1_ = 0.118, *α*_1_ = 1.718, *β*_1_ = 0.029, *μ*_2_ = −0.442, *k*_2_ = 0.079, *α*_2_ = 1.766, *β*_2_ = 0.034, *α*_3_ = 2.138, *β*_3_ = 0.131, *π*_1_ = 0.086, *π*_2_ = 0.071, and *π*_3_ = 1−*π*_1_−*π*_2_ = 0.843. We considered two scenarios to evaluate the effect of sample size on the performance of probe detection. In the first scenario (denoted by G30), we generated 100 datasets using this model, each of which has 1000 genes and 30 pairs of samples. In the second scenario (denoted by G100), we generated 100 datasets using this model, each of which has 1000 genes and 100 pairs of samples.

In the second set of the simulation studies, we generated log_2_ difference of expression levels within a pair of samples using three simple normal distributions, separately. For over-expressed gene probes, we assumed that the log_2_ differences follow $N(\mu_{1},\sigma_{1}^{2})$; for under-expressed gene probes, we assumed that the log_2_ differences follow $N(\mu_{2},\sigma_{2}^{2})$; for non-differentially expressed gene probes, we assumed that the log_2_ differences follow $N(0,\sigma_{3}^{2})$. For simplicity, we set *μ*_1_ = −*μ*_2_ = 2 and *σ*_1_ = *σ*_2_ = 1, *σ*_3_ = 2. In addition, we set the proportion of the over-expressed and under-expressed gene probes as 5 percent respectively. We considered 2 scenarios. In the first scenario (denoted by S30), we generated 100 datasets using this model, each of which has 1000 genes and 30 pairs of samples. In the second scenario (denoted by S100), we generated 100 datasets using this model, each of which has 1000 genes and 100 pairs of samples.

### Existing methods

To best of our knowledge, no existing *MBHM* models could handle data from paired/matched designs. We identified two regularized regression models that could handle data from paired/matched designs [15, 16]. However, we could not find statistical software that implements these two models. Hence, we compared the performance of *eLNNpaired* with several existing hypothesis-based gene selection methods that can handle data from paired/matched design: linear models for microarray data (*limma*) [4], global test (*gt*) [17, 18], significant analysis of microarray (*samr*) [5], and linear model toolset for gene set enrichment analysis (*lmPerGene*) [19]. Using these existing methods, we first performed hypothesis testing for each gene probe, and then adjusted the p-value for multiple testing.

*Limma* and *samr* are essentially paired t-tests with an adjustment for the variance of the mean within-pair difference of gene-expression levels. *Limma* uses probe-specific adjustment based on an empirical Bayesian approach, while *samr* used a fixed constant as adjustment. *Gt* and *lmPerGene* are linear regression approaches, in which the outcome variable measures the within-pair difference of gene expression levels and a non-zero intercept indicates differential gene expression. A positive (negative) test statistic indicates that the gene probe is over (under)-expressed.

For *limma*, *gt*, *samr*, and *lmPerGene*, a gene is detected as differentially expressed if the FDR-adjusted p-value is <0.05. For *samr*, FDR-adjusted p-values were based on 100 permutations.

### Comparison criteria

Five agreement indices and four error rates are used to evaluate the performance of *eLNNpaired*. The five agreement indices are Rand index (*Rand*), Hubert and Arabie’s adjusted Rand index (*HA*), Morey and Agresti’s adjusted Rand index (*MA*), Fowlkes and Mallows’s index (*FM*), and Jaccard index (*Jaccard*) [20]. *HA* and *MA* correct for chance agreement and were recommended by [20]. For perfect agreement, these indices have a value of one. If an index takes a value close to zero (or a negative value), then the agreement between the true probe cluster membership and the estimated probe cluster membership is likely due to chance.

The four error rates are false positive rate (FPR), false negative rate (FNR), false discovery rate (FDR), and false non-discovery rate (FNDR). FPR is the percentage of detected DE probes among truly NE probes. FNR is the percentage of detected NE probes among truly DE probes. FDR is the percentage of truly NE probes among detected DE probes. FNDR is the percentage of truly DE probes among detected NE probes.

For real data sets in which true gene cluster membership is unknown, we applied the Random Forest classification algorithm [21] to predict subjects’ disease statuses based on the detected DE probes and visualized the prediction powers of the 5 probe-detection methods via ROC curves and precision-recall curves.

## Results

We used both real datasets and simulated datasets to evaluate the performance of *eLNNpaired* and to compare its performance with *limma*, *gt*, *samr*, and *lmPerGene*.

### Results for real data

We downloaded from Gene Expression Omnibus (GEO) three gene expression datasets (GSE43292, GSE24742 and GSE6631), all of which used paired designs to collect samples and have been preprocessed by their submitters to ensure good quality of the data. We performed further quality checking to clean the data. (c.f. Section E in S1 File and Figs A, B, and C in S1 File).

For each of the three cleaned GEO datasets, we applied *eLNNpaired*, *limma*, *gt*, *samr*, and *lmPerGene* to identify DE gene probes, which consisted of over-expressed (OE) and under-expressed (UE) genes. For non-differentially expressed gene probes, we denote them by NE. We then used cross table to compare the 3-cluster partitions obtained by the 5 methods (Table 2).

**Table 2:** Cross table of the 3-cluster partitions obtained by *eLNNpaired*, *limma*, *gt*, *samr*, and *lmPerGene*.

		eLNNpaired								
		GSE43292			GSE24742			GSE6631		
		OE	UE	NE	OE	UE	NE	OE	UE	NE
limma	OE	2811	0	1997	0	0	0	772	42	912
	UE	0	2336	2319	0	0	0	0	520	905
	NE	0	0	23834	10	2	54663	0	0	9474
gt	OE	2811	0	1988	0	0	0	772	42	826
	UE	0	2336	2266	0	0	0	0	520	837
	NE	0	0	23896	10	2	54663	0	0	9628
samr	OE	2811	0	3296	0	0	0	772	42	1821
	UE	0	2336	3416	0	0	8	0	520	1790
	NE	0	0	21438	10	2	54655	0	0	7680
lmPerGene	OE	2811	0	2351	10	0	83	772	42	1147
	UE	0	2336	2655	0	2	118	0	520	1174
	NE	0	0	23144	0	0	54462	0	0	8970

**Fig 1:**
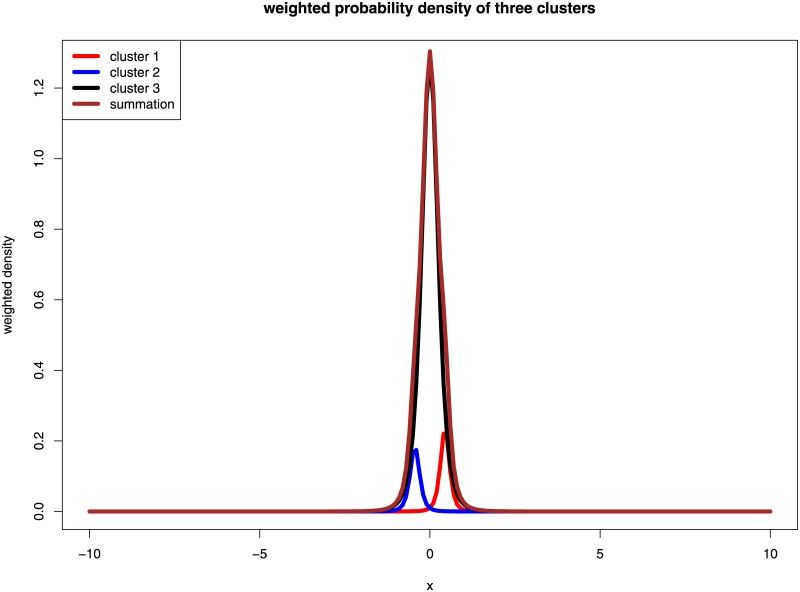
Plots of approximated weighted probability density functions for GSE43292.

**Fig 2:**
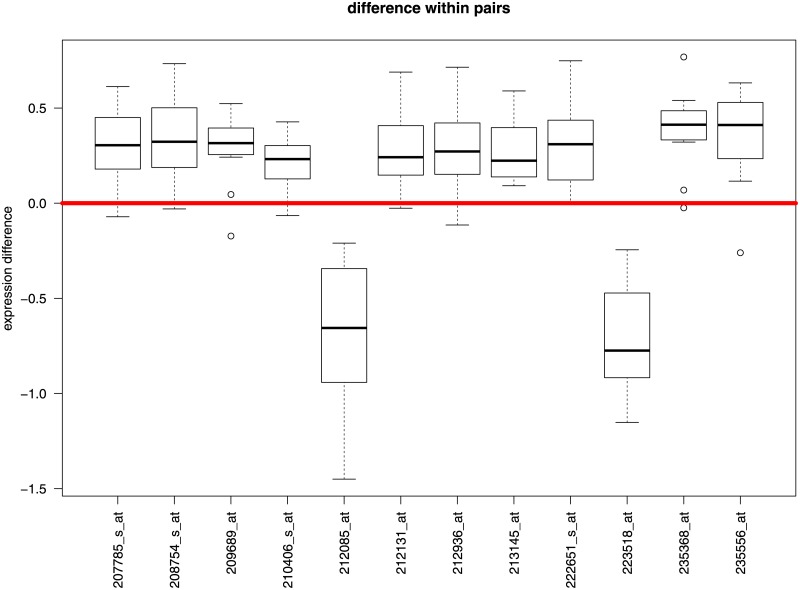
Parallel boxplots of the within-pair log2 difference for the 12 DE probes identified by *eLNNpaired* for GSE24742.

For GSE43292, all gene probes classified as DE (OE or UE) by *eLNNpaired* are in accordance with *limma*. More than 4000 gene probes that were classified as NE by *eLNNpaired* were claimed as OE or UE by *limma*, *gt*, *samr*, and *lmPerGene*. The approximated weighted probability density functions for GSE43292 are presented in Fig 1. For GSE24742 with only 12 pairs of samples, *limma* and *gt* did not identify any DE gene probes, *samr* identified 8 under-expressed gene probes, *lmPerGene* identified 93 over-expressed gene probes and 120 under-expressed gene probes, while *eLNNpaired* identified 10 OE gene probes and 2 UE gene probes. The 8 UE gene probes identified by *samr* were identified as NE by *eLNNpaired*. The 12 DE gene probes identified by *eLNNpaired* were also identified by *lmPerGene*. The parallel boxplots of the within-pair log2 differences across for the 12 DE gene probes identified by *eLNNpaired* demonstrated that the results for GSE24742 by *eLNNpaired* are reasonable (c.f. Fig 2).

For GSE6631, all gene probes classified as OE by *eLNNpaired* are in accordance with *limma*, *gt*, *samr*, and *lmPerGene*; all gene probes, except for 42 gene probes, classified as UE by *eLNNpaired* are in accordance with the other 4 methods. The 1,817 gene probes that were classified as UE by *eLNNpaired* were claimed as OE or UE by *limma*. The 1,663 gene probes that were classified as UE by *eLNNpaired* were claimed as OE or UE by *gt*. The 3,611 gene probes that were classified as UE by *eLNNpaired* were claimed as OE or UE by *samr*. The 2,321 gene probes that were classified as UE by *eLNNpaired* were claimed as OE or UE by *lmPerGene*.

We compared the prediction power of the DE probes obtained by the five probe-detection methods to predict disease statuses of subjects by using the Random Forest algorithm. ROC curves and precision-recall curves are shown in Figs J and K in S1 File. The good performance of all 5 methods was indicated by the fact that all ROC curves were toward the upper-left corner and all precision-recall curves were toward the upper-right corner. Figs J and K in S1 File also indicate that the ROC curve and precision-recall curve of *eLNNpaired* are similar to those of *limma*, *gt*, *samr*, and *lmPerGene*.

The estimates of the *eLNNpaired* model parameters for the 3 GEO datasets are shown in Table 3.

**Table 3:** The estimates of the *eLNNpaired* model parameters for the 3 GEO datasets.

parameter	GSE43292	GSE24742	GSE6631
*π*_1_	0.086	3.36×10^−4^	0.062
*π*_2_	0.071	8.00×10^−5^	0.048
*π*_3_	0.843	0.9996	0.890
*μ*_1_	0.441	0.313	0.502
*k*_1_	0.118	3.572×10^−11^	2.066×10^−10^
*α*_1_	1.718	9.015	2.192
*β*_1_	0.029	0.290	0.111
*μ*_2_	-0.442	-0.672	-0.845
*k*_2_	0.079	7.920×10^−6^	0.503
*α*_2_	1.766	5.042	1.767
*β*_2_	0.034	0.671	0.069
*α*_3_	2.138	0.825	1.393
*β*_3_	0.131	0.551	0.096

### Results for simulation studies

In this section, we evaluated the performance of *eLNNpaired* by two sets of simulation studies. In the first set, the simulated data were generated from the *eLNNpaired* model. The model parameters estimated from GSE43292 were used as the true parameter values. In the second set, the simulated data were *not* generated from *eLNNpaired* model. For each set, we generated 100 simulated datasets, each of which contained expression levels of 1000 gene probes for *n* pairs of samples. To evaluate the effect of sample size, we investigated two scenarios: *n* = 30 pairs and *n* = 100 pairs for each set of simulation studies. We denoted the first set of simulation as G30 and G100 respectively for *n* = 30 and *n* = 100. Similarly, we denoted the second set as S30 and S100 respectively for *n* = 30 and *n* = 100.

**Fig 3:**
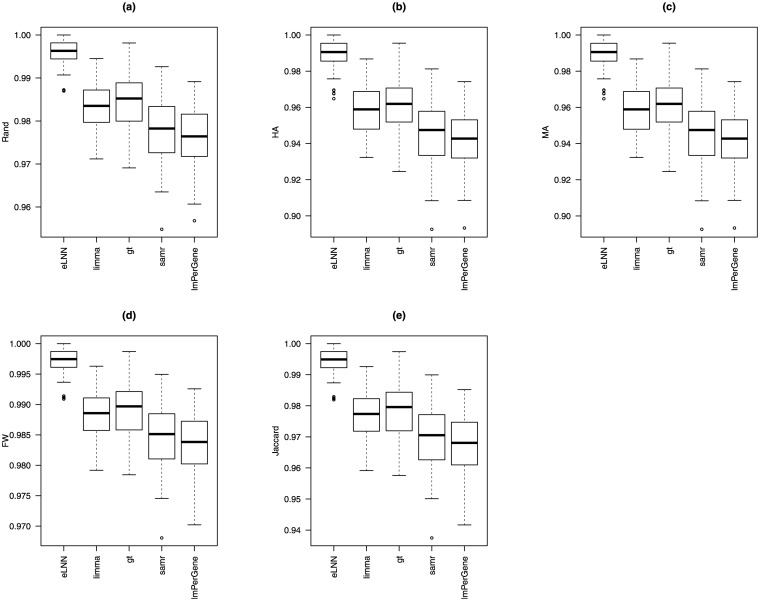
Boxplots of the agreement indices (Rand, HA, MA, FM, and Jaccard) for *eLNNpaired*, *limma*, *gt*, *samr*, and *lmPerGene* based on simulation G30. Top-left panel: Rand; Top-middle panel: HA; Top-right panel: MA; Bottom-left panel: FM; Bottom-right panel: Jaccard.

**Fig 4:**
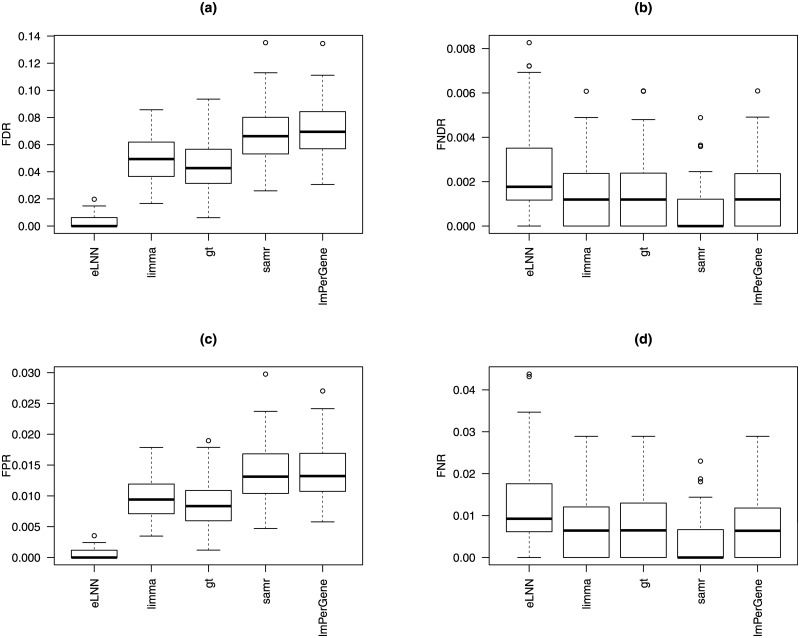
Boxplots of the error rates (FDR, FNDR, FNR, and FPR) for *eLNNpaired*, *limma*, *gt*, *samr*, and *lmPerGene* based on simulation G30. Top-left panel: FDR; Top-right panel: FNDR; Bottom-left panel: FPR; Bottom-right panel: FNR.

Tables 4 and 5 and Tables A and B in S1 File show that all 5 methods had good performance (agreement indices are close to one and error rates are close to zero) for these 2 sets of simulation studies. Figs 3, 4, and Figs D—I in S1 File show that (1) *eLNNpaired* performed better than the other 4 methods in terms of agreement indices, FDR and FPR and (2) *eLNNpaired* had similar FNDR and FNR to *limma*, *gt*, *samr*, and *lmPerGene*.

**Table 4:** Summary of agreement indices from simulation results for the scenario where *n* = 30 pairs per data set.

		eLNNpaired		limma		gt		samr		lmPerGene	
		mean	sd	mean	sd	mean	sd	mean	sd	mean	sd
Rand	G30	0.996	0.003	0.983	0.006	0.984	0.006	0.978	0.007	0.977	0.007
	S30	1.00	0.000	0.989	0.005	0.991	0.004	0.982	0.006	0.983	0.006
HA	G30	0.989	0.008	0.958	0.014	0.961	0.015	0.946	0.017	0.943	0.016
	S30	1.000	0.001	0.965	0.015	0.972	0.013	0.941	0.019	0.946	0.018
MA	G30	0.989	0.008	0.958	0.014	0.961	0.015	0.946	0.017	0.943	0.016
	S30	1.000	0.001	0.965	0.015	0.972	0.013	0.941	0.019	0.946	0.018
FM	G30	0.997	0.002	0.988	0.004	0.989	0.004	0.985	0.005	0.984	0.005
	S30	1.000	0.000	0.993	0.003	0.995	0.003	0.989	0.004	0.990	0.004
Jaccard	G30	0.994	0.004	0.977	0.008	0.978	0.008	0.970	0.010	0.968	0.010
	S30	1.000	0.000	0.987	0.006	0.989	0.005	0.977	0.008	0.980	0.007

**Table 5:** Summary of error rates from simulation results for the scenario where *n* = 30 pairs per data set.

		eLNNpaired		limma		gt		samr		lmPerGene	
		mean	sd	mean	sd	mean	sd	mean	sd	mean	sd
FDR	G30	0.003	0.005	0.049	0.017	0.045	0.018	0.068	0.021	0.069	0.020
	S30	0.000	0.002	0.054	0.023	0.044	0.020	0.088	0.027	0.081	0.026
FNDR	G30	0.002	0.002	0.001	0.001	0.002	0.001	0.001	0.001	0.001	0.001
	S30	0.000	0.000	0.000	0.000	0.000	0.000	0.000	0.000	0.000	0.000
FPR	G30	0.001	0.001	0.010	0.003	0.009	0.004	0.014	0.005	0.014	0.004
	S30	0.000	0.000	0.006	0.003	0.005	0.002	0.011	0.004	0.010	0.003
FNR	G30	0.012	0.010	0.007	0.007	0.008	0.007	0.004	0.005	0.007	0.007
	S30	0.000	0.000	0.000	0.000	0.000	0.000	0.000	0.000	0.000	0.000

## Discussion

In this paper, we aimed to extend existing *MBHM* methods to analyze genomic data collected from paired/matched designs. The proposed model does not involve hypothesis testing; hence it does not have the problem of the curse of dimensionality. The proposed model can also borrow information across genes to estimate hyper-parameters, which makes it useful for data with small sample sizes.

The performance of the proposed model in detecting DE gene probes worked better than the existing hypothesis-based methods *limma*, *gt*, *samr*, and *lmPerGene* in terms of agreement indices in the simulation studies.

Both simulation studies and real data analyses showed that the proposed model had similar error rates and prediction accuracy to *limma*, *gt*, *samr*, and *lmPerGene*, although the proposed model detected much fewer DE probes than the other four methods.

One advantage of the proposed model over the existing *MBHM* methods is that it introduces constraints on the model hyper-parameters to reduce false discoveries. More stringent constraints could result in fewer positives and a reduction in false discoveries. One possible benefit of the constraint setting is to make it adaptive to different datasets. Specifically, we can first set constraints empirically, then compare the derived results with what *limma* provides. If a gene is classified by *limma* as over-expressed but by our model as under-expressed or the other way round, we assume that *limma* is correct and tighten our constraints by a small amount. If no such genes are discovered, we loosen our constraints in the same manner. Under these new constraints, we run our model again. We repeat this procedure until we reach a critical point where we find as many positives as possible, while also avoiding false discoveries to a large extent.

For example, for GSE6631, 42 genes were classified as OE by our model, but as UE by *limma*. This can be reduced by setting the constraints stronger. For instance, we can set *c*_2_ = Φ^−1^(0.025) instead of Φ^−1^(0.05), and we will get a new cross table with less number of false UE (c.f. Table 6).

**Table 6:** New cross table for GSE6631.

		eLNNpaired		
		OE	UE	NE
limma	OE	773	2	951
	UE	0	412	1013
	NE	0	0	9474

Since the parameter estimation of the proposed model is based on the EM algorithm, which is computationally inefficient, the adaptive constraints introduced above may take too much time. One efficient way to reduce false discovery is to compare the results with what *limma* provides, and use *limma*’s result when conflicts are found between over-expressed and under-expressed genes.

It is well known that the EM algorithm converges slowly. Based on the Appendix E of [22], the computational complexity of one EM iteration is $O(nG+KG^{2})$, where *n* is the number of sample pairs, *G* is the number of gene probes, and *K* = 3 is the number of mixtures. We used R language to implement the *eLNNpaired* algorithm, and this produced results in reasonable time. For example, we used a Linux Machine running 64-bit CentOS 6.8 Linux with 4 cores, 24G memory, 2.6 GHz Xeon CPU and the running times for the 3 GEO data sets are listed in Table 7. In the future, we can use FORTRAN language to program the core parts of the *eLNNpaired* algorithm and then use R to call the FORTRAN functions to improve the speed of *eLNNpaired*.

**Table 7:** Total elapsed times (seconds) for the 3 GEO data sets.

	GSE43292	GSE24742	GSE6631
eLNNpaired	139.679	170.833	61.561
limma	5.325	6.619	2.720
gt	326.308	553.442	128.067
samr	47.709	63.136	20.737
lmPerGene	1.082	1.563	0.482

The proposed method can be used or adapted for analyzing other types of omics data, such as DNA methylation data, microRNA data, metabolite data, or next generation sequencing data.

The proposed model has some limitations. First, the model, like other MBHMs, assumes that gene probes are independent, which could not be totally satisfied by the real data since physically adjacent gene probes might be positively correlated. Ignoring positive correlation would typically reduce the number of positive test results. Since the proposed model borrows information across genes, this may counter the effects of ignoring positive correlation. Future research is warranted to study how to incorporate gene-gene correlation into our model.

To simplify the model building and parameter estimation, we assume that the within-pair log_2_ difference of expression levels is conditional normally distributed and we impose conjugate prior distributions on model parameters. Real data analysis usually shows that conditional normality assumption is reasonable. In further work, we will experiment with other priors or a non-informative prior and use Bayesian estimation (e.g., Markov Chain Monte Carlo (MCMC) method) to estimate model hyper-parameters.

We implemented the *eLNNpaired* algorithm to an R package (S2 File), which is freely available to researchers.

## Supporting information

S1 File Supplementary documents. Existing MBHMs; The marginal distributions; Objective function in M-step; Initialization of EM algorithm; GEO data QC plots; Simulation results for scenarios with 100 pairs of samples; Comparing the power of the significant probes to predict disease status.
(PDF)

S2 File The tarball file for the R package that implements the *eLNNpaired* algorithm: eLNNpaired_0.2.2.tar.gz. (GZ)

## References

[1] Bolstad BM, Irizarry RA, Åstrand M, Speed TP. A comparison of normalization methods for high density oligonucleotide array data based on variance and bias. Bioinformatics. 2003;19(2):185–193. 10.1093/bioinformatics/19.2.185 12538238

[2] Leek JT, Storey JD. Capturing heterogeneity in gene expression studies by surrogate variable analysis. PLoS Genet. 2007;3(9):1724–1735. 10.1371/journal.pgen.0030161 17907809 PMC1994707

[3] Huber W, Carey VJ, Gentleman R, et al Orchestrating high-throughput genomic analysis with Bioconductor. Nature Methods. 2015;12(2):115–121. 10.1038/nmeth.3252 25633503 PMC4509590

[4] Smyth GK. Linear models and empirical Bayes methods for assessing differential expression in microarray experiments. Statistical Applications in Genetics and Molecular Biology. 2004;3:Article3 10.2202/1544-6115.1027 16646809

[5] Tusher V G, Tibshirani R, Chu G. Significance analysis of microarrays applied to the ionizing radiation response. Proceedings of the National Academy of Sciences. 2001;98(9):5116–5121. 10.1073/pnas.091062498 PMC33173 11309499

[6] Tibshirani R. Regression shrinkage and selection via the lasso. Journal of the Royal Statistical Society Series B (Methodological). 1996; p. 267–288.

[7] Wu B. Differential gene expression detection using penalized linear regression models: the improved SAM statistics. Bioinformatics. 2005;21(8):1565–1571. 10.1093/bioinformatics/bti217 15598833

[8] Newton MA, Kendziorski CM, Richmond CS, Blattner FR, Tsui KW. On differential variability of expression ratios: improving statistical inference about gene expression changes from microarray data. Journal of computational biology. 2001;8(1):37–52. 10.1089/106652701300099074 11339905

[9] Kendziorski CM, Newton MA, Lan H, Gould MN. On parametric empirical Bayes methods for comparing multiple groups using replicated gene expression profiles. Statistics in medicine. 2003;22(24):3899–3914. 10.1002/sim.1548 14673946

[10] Lo K, Gottardo R. Flexible empirical Bayes models for differential gene expression. Bioinformatics. 2007;23:328–335. 10.1093/bioinformatics/btl612 17138586

[11] Qiu WL, He W, Wang X, Lazarus R. A Marginal Mixture Model for Selecting Differentially Expressed Genes across Two Types of Tissue Samples. International Journal of Biostatistics. 2008;4(1):20 10.2202/1557-4679.1093 PMC2835454 20231912

[12] Ayari H, Bricca G. Identification of two genes potentially associated in iron-heme homeostasis in human carotid plaque using microarray analysis. Journal of biosciences. 2013;38(2):311–315. 10.1007/s12038-013-9310-2 23660665

[13] Gutierrez-Roelens I, Galant C, Theate I, Lories RJ, Durez P, Nzeusseu-Toukap A, et al Rituximab treatment induces the expression of genes involved in healing processes in the rheumatoid arthritis synovium. Arthritis & Rheumatism. 2011;63(5):1246–1254. 10.1002/art.30292 21337318

[14] Kuriakose MA, Chen WT, He ZM, Sikora AG, Zhang P, Zhang ZY, et al Selection and validation of differentially expressed genes in head and neck cancer. Cellular and Molecular Life Sciences CMLS. 2004;61(11):1372–1383. 10.1007/s00018-004-4069-0 15170515 PMC11138891

[15] Avalos M, Pouyes H, Grandvalet Y, Orriols L, Lagarde E. Sparse conditional logistic regression for analyzing large-scale matched data from epidemiological studies: a simple algorithm. BMC Bioinformatics. 2013;16 (Suppl 6):S1 10.1186/1471-2105-16-S6-S1 PMC4416185 25916593

[16] Qian J, Payabvash S, Kemmling A, Lev MH, Schwamm LH, Betensky RA. Variable Selection and Prediction Using a Nested, Matched Case-Control Study: Application to Hospital Acquired Pneumonia in Stroke Patients. Biometrics. 2014;70(1):153–163. 10.1111/biom.12113 24320930 PMC3954429

[17] Goeman J J, van de Geer S A, de Kort F, van Houwelingen H C. A global test for groups of genes: testing association with a clinical outcome. Bioinformatics. 2004;20:93–99. 10.1093/bioinformatics/btg382 14693814

[18] Goeman J J, van de Geer S A, van Houwelingen H C. Testing against a high-dimensional alternative. Journal of the Royal Statistical Society, Series B. 2006;68:477–493. 10.1111/j.1467-9868.2006.00551.x

[19] Oron A, Jiang Z, Gentleman R. Gene set enrichment analysis using linear models and diagnostics. Bioinformatics. 2008;24:2586–2591. 10.1093/bioinformatics/btn465 18790795 PMC2579710

[20] Milligan G W, Cooper M C. A study of the comparability of external criteria for hierarchical cluster analysis. Multivariate Behavioral Research. 1986;21:441–458. 10.1207/s15327906mbr2104_5 26828221

[21] Breiman L. Random Forests. Machine Learning. 2001;45(1):5–32. 10.1023/A:1010933404324

[22] Chen Z, Haykin S, Eggermont J J, Becker S. Correlative Learning: A Basis for Brain and Adaptive Systems. John Wiley & Sons, Inc; 2007.

