# Supplementary material for: Detecting disease-associated genomic outcomes using constrained mixture of Bayesian hierarchical models for paired data
Source: PLoS One. 2017 Mar 30;12(3):e0174602. doi: 10.1371/journal.pone.0174602 (PMC5373614; doi:10.1371/journal.pone.0174602)
Supplement: S1 File — Existing MBHMs; The marginal distributions; Objective function in M-step; Initialization of EM algorithm; GEO data QC plots; Simulation results for scenarios with 100 pairs of samples; Comparing the power of the significant probes to predict disease status. (PDF) [file pone.0174602.s001.pdf]

Supplementary Documents

Yunfeng Li[1], Jarrett Morrow[2], Benjamin Raby[2], Kelan Tantisira[2], Scott T. Weiss[2], Wei Huang[1], Weiliang Qiu[2*]

[1]: School of Mathematical Sciences, Zhejiang University, HangZhou, Zhejiang, China

[2]: Channing Division of Network Medicine, Brigham and Women's Hospital/Harvard Medical School, Boston, MA USA

A Existing MBHMs

Existing MBHMs include Gamma-Gamma model (GG)[3], LogNormal-Normal model (LNN)[1], extended Gamma-Gamma model (eGG)[2], and extended LogNormal-Normal model (eLNN)[2]. In the followings, we briefly describe the eLNN model. For details of eLNN and other models, please refer to the corresponding references. All these 4 models assume that samples are independent.

The eLNN model assumes that there are 2 clusters of gene probes: (1) gene probes non-differentially expressed (NE) between cases and controls and (2) gene probes differentially expressed (DE) between cases and controls. Let

$$\boldsymbol{Z}_g = \begin{pmatrix} Z_{g,1} \\ Z_{g,2} \\ \vdots \\ Z_{g,m_c} \\ Z_{g,m_c+1} \\ Z_{g,m_c+2} \\ \vdots \\ Z_{g,m_c+m_n} \end{pmatrix} \tag{A1}$$

denote the gene expression profile for the g-th gene across two different types of tissues with m_c and m_n tissues, respectively. Without loss of generality, we assume the first m_c elements of $\boldsymbol{Z}_g$ correspond to the first type of tissue samples (cancer tissue samples) and the second m_n elements of $\boldsymbol{Z}_g$ correspond to the second type of tissue samples (normal tissue samples).

For the g-th NE gene probe, the eLNN model assumes the following Bayesian hierarchical model

$$\begin{aligned} \log\left(Z_{g,\ell}\right)|\mu_g, \tau_g^{-1} &\sim \mathrm{N}\left(\mu_g, \tau_g^{-1}\right), \ell = 1, \ldots, m_c + m_n \\ \mu_g|\tau_g^{-1} &\sim \mathrm{N}\left(\mu_0, k\tau_g^{-1}\right) \\ \tau_g &\sim \mathrm{Gamma}(\alpha, 1/\beta) \end{aligned} \tag{A2}$$

where α is the shape parameter and β is the rate parameter, or equivalently, $1/\beta$ is the scale parameter.

For the g-th DE gene probe, the eLNN model assumes the following Bayesian hierarchical model

$$\begin{aligned} \log\left(Z_{g,\ell}\right)|\mu_{g,c}, \tau_{g,c}^{-1} &\sim \mathrm{N}\left(\mu_{g,c}, \tau_{g,c}^{-1}\right), \ell = 1, \ldots, m_c \\ \mu_{g,c}|\tau_{g,c}^{-1} &\sim \mathrm{N}\left(\mu_0, k\tau_{g,c}^{-1}\right) \\ \tau_{g,c} &\sim \mathrm{Gamma}(\alpha, 1/\beta) \\ \log\left(Z_{g,\ell'}\right)|\mu_{g,n}, \tau_{g,n}^{-1} &\sim \mathrm{N}\left(\mu_{g,n}, \tau_{g,n}^{-1}\right), \ell' = m_c + 1, \ldots, m_c + m_n \\ \mu_{g,n}|\tau_{g,n}^{-1} &\sim \mathrm{N}\left(\mu_0, k\tau_{g,n}^{-1}\right) \\ \tau_{g,n} &\sim \mathrm{Gamma}(\alpha, 1/\beta) \end{aligned} \tag{A3}$$

For the g-th NE gene probe, its marginal distribution of gene expression levels $\boldsymbol{Z}_g$ is

$$\begin{aligned} f(\boldsymbol{Z}_g|\mu_0, k, \alpha, \beta) &\overset{H_0}{=} \int\int f(\boldsymbol{Z}_g, \mu_g, \tau_g^{-1}) d\mu_g d\tau_g^{-1} \\ &= \int\int f(\boldsymbol{Z}_g|\mu_g, \tau_g^{-1}) f(\mu_g, \tau_g^{-1}) d\mu_g d\tau^{-1} d\mu_g d\tau_g^{-1} \\ &= \int\int \left[\prod_{\ell}^{m_c+m_n} f(Z_{g,\ell}|\mu_g, \tau_g^{-1})\right] f(\mu_g, \tau_g^{-1}) d\mu_g d\tau^{-1} d\mu_g d\tau_g^{-1}. \end{aligned} \tag{A4}$$

The last equation is due to the assumption that $Z_{g,\ell}$ given μ_g, τ_g^{-1}, $\ell = 1, \ldots, m_c + m_n$, are conditionally independent.

Similarly, for the g-th DE gene probe, its marginal distribution of $\boldsymbol{Z}_g$ is

$$
\begin{aligned}
f(\boldsymbol{Z}_g|\mu_0, k, \alpha, \beta) &\overset{H_a}{=} \int\int f(\boldsymbol{Z}_g, \mu_{g,c}, \tau_{g,c}^{-1}, \mu_{g,n}, \tau_{g,n}^{-1}) d\mu_{g,c} d\tau_{g,c}^{-1} d\mu_{g,n} d\tau_{g,n}^{-1} \\
&= \int\int f(\boldsymbol{Z}_g|\mu_{g,c}, \tau_{g,c}^{-1}, \mu_{g,n}, \tau_{g,n}^{-1}) f(\mu_{g,c}, \tau_{g,c}^{-1}, \mu_{g,n}, \tau_{g,n}^{-1}) d\mu_{g,c} d\tau_{g,c}^{-1} d\mu_{g,n} d\tau_{g,n}^{-1} \\
&= \int\int \left[\prod_{\ell=1}^{m_c} f(Z_{g,\ell}|\mu_{g,c}, \tau_{g,c}^{-1})\right] f(\mu_{g,c}, \tau_{g,c}^{-1}) d\mu_{g,c} d\tau_{g,c}^{-1} \\
&\quad \cdot \int\int \left[\prod_{\ell'=m_c+1}^{m_c+m_n} f(Z_{g,\ell'}|\mu_{g,n}, \tau_{g,n}^{-1})\right] f(\mu_{g,n}, \tau_{g,n}^{-1}) d\mu_{g,n} d\tau_{g,n}^{-1}.
\end{aligned}
\tag{A5}
$$

B The marginal distributions of eLNNpaired

For simplicity, we use the original parameters, rather than re-parameterized parameters, in the following formulae.

In the derivation of the marginal distributions, we used the following facts about the Pearson type VII distribution and the property of a probability density function.

The density of the Pearson type VII distribution (symmetric 3-parameter family) is

$$
f(x) = \frac{1}{\xi B\left(m - \frac{1}{2}, \frac{1}{2}\right)\left[1 + \left(\frac{x-\lambda}{\xi}\right)^2\right]^m}, \tag{A6}
$$

where B is the Beta function

$$
B(x, y) = \int_0^1 t^{x-1}(1-t)^{y-1} dt, \tag{A7}
$$

and

$$B(x,y) = \frac{\Gamma(x)\Gamma(y)}{\Gamma(x+y)}. \tag{A8}$$

$$\Gamma(1/2) = \sqrt{\pi}. \tag{A9}$$

If $f(x)$ is a density function, then

$$\int f(x)dx = 1. \tag{A10}$$

The marginal distribution for over-expressed (OE) gene probes is

$$\begin{aligned}
&f_1(\mathbf{d}_g|\boldsymbol{\psi}) \\
=&\int_0^\infty \int_{-\infty}^\infty \left[\prod_l \mathrm{N}(d_{gl};\mu_g,\tau_g^{-1})\right] \mathrm{N}(\mu_g;\mu_1,k_1\tau_g^{-1})\mathrm{G}(\tau_g;\alpha_1,\beta_1)d\mu_g\tau_g \\
=&\int_0^\infty \int_{-\infty}^\infty \left(\frac{\sqrt{\tau_g}}{\sqrt{2\pi}}\right)^n e^{-\frac{\tau_g}{2}\left(\sum_l(d_{gl}-\mu_g)^2\right)} \frac{\sqrt{\tau_g}}{\sqrt{2\pi k_1}} e^{-\frac{\tau_g}{2k_1}(\mu_g-\mu_1)^2} \frac{\beta_1^{\alpha_1}}{\Gamma(\alpha_1)}\tau_g^{\alpha_1-1}e^{-\beta_1\tau_g}d\mu_g\tau_g \\
=&\frac{\beta_1^{\alpha_1}}{\Gamma(\alpha_1)(\sqrt{2\pi})^n}\int_0^\infty \int_{-\infty}^\infty \tau_g^{n/2+\alpha_1-1}e^{-\beta_1\tau_g}\frac{\sqrt{\tau_g}}{\sqrt{2\pi k_1}}e^{-\frac{\tau_g}{2k_1}\left(\sum_l(k_1(d_{gl}-\mu_g)^2)+(\mu_g-\mu_1)^2\right)}d\mu_g\tau_g \\
=&\frac{\beta_1^{\alpha_1}}{\Gamma(\alpha_1)(\sqrt{2\pi})^n}\int_0^\infty \int_{-\infty}^\infty \tau_g^{n/2+\alpha_1-1}e^{-\beta_1\tau_g}\frac{1}{\sqrt{nk_1+1}}e^{-\frac{\tau_g}{2k_1}\left(k_1\sum_l d_{gl}^2+\mu_1^2-\frac{(k_1\sum_l d_{gl}+\mu_1)^2}{nk_1+1}\right)} \\
&\times\frac{\sqrt{\tau_g(nk_1+1)}}{\sqrt{2\pi k_1}}e^{-\frac{\tau_g(nk_1+1)}{2k_1}\left(\mu_g-\frac{k_1\sum_l d_{gl}+\mu_1}{nk_1+1}\right)^2}d\mu_g\tau_g \\
=&\frac{\beta_1^{\alpha_1}}{\Gamma(\alpha_1)(\sqrt{2\pi})^n}\int_0^\infty \tau_g^{n/2+\alpha_1-1}e^{-\beta_1\tau_g}\frac{1}{\sqrt{nk_1+1}}e^{-\frac{\tau_g}{2k_1}\left(k_1\sum_l d_{gl}^2+\mu_1^2-\frac{(k_1\sum_l d_{gl}+\mu_1)^2}{nk_1+1}\right)}d\tau_g \\
=&\frac{\beta_1^{\alpha_1}\Gamma(\frac{n}{2}+\alpha_1)}{\Gamma(\alpha_1)(2\pi)^{n/2}\sqrt{nk_1+1}}\left[\beta_1+\frac{1}{2k_1}\left(k_1\sum_l d_{gl}^2+\mu_1^2-\frac{(k_1\sum_l d_{gl}+\mu_1)^2}{nk_1+1}\right)\right]^{-(\frac{n}{2}+\alpha_1)} \\
=&\frac{\beta_1^{\alpha_1}\Gamma(\frac{n}{2}+\alpha_1)}{\Gamma(\alpha_1)(2\pi)^{n/2}\sqrt{nk_1+1}} \\
&\times\left[\beta_1+\frac{1}{2}\sum_l d_{gl}^2-\frac{1}{2n}\left(\sum_l d_{gl}\right)^2+\frac{n}{2(nk_1+1)}\left(\mu_1-\frac{\sum_l d_{gl}}{n}\right)^2\right]^{-(\frac{n}{2}+\alpha_1)}
\end{aligned} \tag{A11}$$

The marginal distribution for under-expressed (UE) gene probes can be derived similarly

$$
\begin{aligned}
&f_2(\mathbf{d}_g|\boldsymbol{\psi}) \\
=&\int_0^\infty \int_{-\infty}^\infty \left[\prod_l \mathrm{N}(d_{gl};\mu_g,\tau_g^{-1})\right] \mathrm{N}(\mu_g;\mu_2,k_2\tau_g^{-1})\mathrm{G}(\tau_g;\alpha_2,\beta_2)d\mu_g\tau_g \\
=&\frac{\beta_2^{\alpha_2}\Gamma(\frac{n}{2}+\alpha_2)}{\Gamma(\alpha_2)(2\pi)^{n/2}\sqrt{nk_2+1}} \\
&\times \left[\beta_2 + \frac{1}{2}\sum_l d_{gl}^2 - \frac{1}{2n}\left(\sum_l d_{gl}\right)^2 + \frac{n}{2(nk_2+1)}\left(\mu_2 - \frac{\sum_l d_{gl}}{n}\right)^2\right]^{-(\frac{n}{2}+\alpha_2)}
\end{aligned}
\tag{A12}
$$

The marginal distribution for non-differentially expressed (NE) gene probes is simpler and has the form:

$$
\begin{aligned}
&f_3(\mathbf{d}_g|\boldsymbol{\psi}) \\
=&\int_0^\infty \left[\prod_l \mathrm{N}(d_{gl};0,\tau_g^{-1})\right] \mathrm{G}(\tau_g;\alpha_3,\beta_3)\tau_g \\
=&\frac{\beta_3^{\alpha_3}\Gamma(\frac{n}{2}+\alpha_3)}{\Gamma(\alpha_3)(2\pi)^{n/2}}\left(\beta_3 + \frac{1}{2}\sum_l d_{gl}^2\right)^{-(\frac{n}{2}+\alpha_3)}
\end{aligned}
\tag{A13}
$$

C The EM algorithm

With the help of indicator variables $\mathbf{z}_g$ and Dirichlet prior $D(\boldsymbol{b})$, we have the likelihood function

$$\begin{aligned} &L(\boldsymbol{\pi}, \boldsymbol{\psi}|\mathbf{d}, \mathbf{z}) \\ =& \left(\prod_{g=1}^{G} f(\mathbf{d}_g|\mathbf{z}_g, \boldsymbol{\psi}, \boldsymbol{\pi})\right) \mathrm{Dir}(\boldsymbol{b}) \\ =& \left(\prod_{g=1}^{G} (\pi_1 f_1(\mathbf{d}_g|\boldsymbol{\psi}))^{z_{g1}} (\pi_2 f_2(\mathbf{d}_g|\boldsymbol{\psi}))^{z_{g2}} (\pi_3 f_3(\mathbf{d}_g|\boldsymbol{\psi}))^{z_{g3}}\right) \frac{\Gamma(\sum_{c=1}^{3} b_c)}{\prod_{c=1}^{3}\Gamma(b_c)} \prod_{c=1}^{3} \pi_c^{b_c-1}. \end{aligned} \tag{A14}$$

and correspondingly, the log-likelihood function

$$\begin{aligned} &l(\boldsymbol{\pi}, \boldsymbol{\psi}|\mathbf{d}, \mathbf{z}) \\ =& \sum_g (z_{g1} \log f_1(\mathbf{d}_g|\boldsymbol{\psi}) + z_{g2} \log f_2(\mathbf{d}_g|\boldsymbol{\psi}) + z_{g3} \log f_3(\mathbf{d}_g|\boldsymbol{\psi})) \\ &+ \sum_g (z_{g1} \log \pi_1 + z_{g2} \log \pi_2 + z_{g3} \log \pi_3) \\ &+ \log\left(\frac{\Gamma(\sum_{c=1}^{3} b_c)}{\prod_{c=1}^{3}\Gamma(b_c)}\right) + \sum_{c=1}^{3}(b_c - 1)\log \pi_c. \end{aligned} \tag{A15}$$

In the E-step, for the g-th gene probe, we denote by $\tilde{z}_{g1}$ the probability of $\mathbf{z}_g = (1, 0, 0)$, $\tilde{z}_{g2}$ the probability of $\mathbf{z}_g = (0, 1, 0)$ and $\tilde{z}_{g3}$ the probability of $\mathbf{z}_g = (0, 0, 1)$. We denote these

three variables by $\tilde{\mathbf{z}}_g = (\tilde{z}_{g1}, \tilde{z}_{g2}, \tilde{z}_{g3})$. Applying Bayes rule, we obtain:

$$\begin{aligned}\tilde{z}_{gc} &= \Pr(z_{gc} = 1|\mathbf{d}_g, \boldsymbol{\pi}, \boldsymbol{\psi}) \\ &= \frac{\Pr(\mathbf{d}_g|g \text{ is in cluster } c)\Pr(g \text{ is in cluster } c)}{\sum_c \Pr(\mathbf{d}_g|g \text{ is in cluster } c)\Pr(g \text{ is in cluster } c)} \\ &= \frac{\pi_c f_c(\mathbf{d}_g|\boldsymbol{\psi})}{\pi_1 f_1(\mathbf{d}_g|\boldsymbol{\psi}) + \pi_2 f_2(\mathbf{d}_g|\boldsymbol{\psi}) + \pi_3 f_3(\mathbf{d}_g|\boldsymbol{\psi})},\end{aligned} \tag{A16}$$

for $c = 1, 2, 3$.

Since $\mathbf{z}_g$'s are unknown, we integrate out $\mathbf{z}_g$ and get the expected log likelihood function:

$$\begin{aligned}&Q(\boldsymbol{\pi}, \boldsymbol{\psi}|\mathbf{d}) \\ =&\sum_g (\tilde{z}_{g1} \log f_1(\mathbf{d}_g|\boldsymbol{\psi}) + \tilde{z}_{g2} \log f_2(\mathbf{d}_g|\boldsymbol{\psi}) + \tilde{z}_{g3} \log f_3(\mathbf{d}_g|\boldsymbol{\psi})) \\ &+ \sum_g (\tilde{z}_{g1} \log \pi_1 + \tilde{z}_{g2} \log \pi_2 + \tilde{z}_{g3} \log \pi_3) \\ &+ \log \left(\frac{\Gamma(\sum_{c=1}^3 b_c)}{\prod_{c=1}^3 \Gamma(b_c)} \right) + \sum_{c=1}^3 (b_c - 1) \log \pi_c.\end{aligned} \tag{A17}$$

In the M-step, we maximize the log likelihood over parameters $\boldsymbol{\pi}$ and $\boldsymbol{\psi}$. We used the built-in function *optim* of the statistical software *R* and its *L-BFGS-B* optimization procedure to do the maximization.

In the followings, we derive the gradients of Q. Since $\pi_1 + \pi_2 + \pi_3 = 1$, we can eliminate π_3 by $\pi_3 = 1 - \pi_1 - \pi_2$. It is also worthy to point out the fact that $\sum_g (\tilde{z}_{g1} + \tilde{z}_{g2} + \tilde{z}_{g3}) = G$. We take partial derivative of Q w.r.t π_1 and π_2:

$$\frac{\partial Q}{\partial \pi_1} = \sum_g \left(\frac{\tilde{z}_{g1}}{\pi_1} - \frac{\tilde{z}_{g3}}{1 - \pi_1 - \pi_2} \right) + \frac{b_1 - 1}{\pi_1} - \frac{b_3 - 1}{1 - \pi_1 - \pi_2}, \tag{A18}$$

$$\frac{\partial Q}{\partial \pi_2} = \sum_g \left(\frac{\tilde{z}_{g2}}{\pi_2} - \frac{\tilde{z}_{g3}}{1 - \pi_1 - \pi_2} \right) + \frac{b_2 - 1}{\pi_2} - \frac{b_3 - 1}{1 - \pi_1 - \pi_2} \tag{A19}$$

where $\boldsymbol{b} = (b_1, b_2, b_3)$ is the parameter for Dirichlet prior. Setting $b_1 = b_2 = b_3 = 2$, we can solve the equations for $\boldsymbol{\pi}$:

$$\pi_1 = \frac{\sum_g \tilde{z}_{g1} + 1}{G + 3}, \pi_2 = \frac{\sum_g \tilde{z}_{g2} + 1}{G + 3}, \pi_3 = \frac{\sum_g \tilde{z}_{g3} + 1}{G + 3}. \tag{A20}$$

We derive the Hessian matrix to show Q reaches its maxima w.r.t. $\boldsymbol{\pi}$:

$$\frac{\partial^2 Q}{\partial \pi_1^2} = -\sum_g \left(\frac{\tilde{z}_{g1}}{\pi_1^2} + \frac{\tilde{z}_{g3}}{(1 - \pi_1 - \pi_2)^2} \right) - \frac{1}{\pi_1^2} - \frac{1}{(1 - \pi_1 - \pi_2)^2},$$

$$\frac{\partial^2 Q}{\partial \pi_1 \partial \pi_2} = -\sum_g \left(\frac{\tilde{z}_{g3}}{(1 - \pi_1 - \pi_2)^2} \right) - \frac{1}{(1 - \pi_1 - \pi_2)^2},$$

$$\frac{\partial^2 Q}{\partial \pi_2 \partial \pi_1} = -\sum_g \left(\frac{\tilde{z}_{g3}}{(1 - \pi_1 - \pi_2)^2} \right) - \frac{1}{(1 - \pi_1 - \pi_2)^2},$$

$$\frac{\partial^2 Q}{\partial \pi_2^2} = -\sum_g \left(\frac{\tilde{z}_{g2}}{\pi_1^2} + \frac{\tilde{z}_{g3}}{(1 - \pi_1 - \pi_2)^2} \right) - \frac{1}{\pi_2^2} - \frac{1}{(1 - \pi_1 - \pi_2)^2}.$$

So for any $\boldsymbol{x} = (x_1, x_2)' \neq (0,0)'$, we have:

$$
\begin{aligned}
&\boldsymbol{x}' \begin{bmatrix} \frac{\partial^2 Q}{\partial \pi_1^2} & \frac{\partial^2 Q}{\partial \pi_1 \partial \pi_2} \\ \frac{\partial^2 Q}{\partial \pi_2 \partial \pi_1} & \frac{\partial^2 Q}{\partial \pi_2^2} \end{bmatrix} \boldsymbol{x} \\
=& -\left(\frac{\sum_g \tilde{z}_{g1}}{\pi_1^2} + \frac{1}{\pi_1^2} \right) x_1^2 - \left(\frac{\sum_g \tilde{z}_{g2}}{\pi_2^2} + \frac{1}{\pi_2^2} \right) x_2^2 \\
&- \left(\frac{\sum_g \tilde{z}_{g3}}{(1-\pi_1-\pi_2)^2} + \frac{1}{(1-\pi_1-\pi_2)^2} \right) (x_1 + x_2)^2 \\
<& 0.
\end{aligned} \tag{A21}
$$

This indicates that the Hessian matrix is negative definite.

For the rest derivatives, we list them by clusters:

1. w.r.t. $\mu_1, k_1, \alpha_1, \beta_1$: Denote

$$
\begin{aligned}
A_1 =& \frac{n}{2(nk_1+1)}, \\
B_{1g} =& \frac{\sum_l d_{gl}}{n}, \\
C_{1g} =& \beta_1 + \frac{1}{2} \sum_l d_{gl}^2 - \frac{\left(\sum_l d_{gl}\right)^2}{2n}, \\
D_1 =& \alpha_1 \log \beta_1 + \log \Gamma\left(\frac{n}{2} + \alpha_1\right) - \log \Gamma(\alpha_1) - \frac{n}{2} \log(2\pi) - \frac{1}{2} \log(nk_1 + 1).
\end{aligned}
$$

Then we have

$$
\frac{\partial Q}{\partial \mu_1} = -\left(\frac{n}{2} + \alpha_1\right) \sum_g \left(\tilde{z}_{g1} \frac{2A_1(\mu_1 - B_{1g})}{A_1(\mu_1 - B_{1g})^2 + C_{1g}} \right),
$$

$$
\frac{\partial Q}{\partial k_1} = -\frac{n \sum_g \tilde{z}_{g1}}{2(nk_1+1)} + \frac{n^2}{2(nk_1+1)^2} \left(\frac{n}{2} + \alpha_1\right) \sum_g \frac{\tilde{z}_{g1}(\mu_1 - B_{1g})^2}{A(\mu_1 - B_{1g})^2 + C_{1g}},
$$

$$\frac{\partial Q}{\partial \alpha_1} = \sum_g \left(\log \beta_1 + \frac{\Gamma'(\frac{n}{2} + \alpha_1)}{\Gamma(\frac{n}{2} + \alpha_1)} - \frac{\Gamma'(\alpha_1)}{\Gamma(\alpha_1)} \right) - \sum_g \left(\tilde{z}_{g1} \log \left(A_1 \left(\mu_1 - B_{1g} \right)^2 + C_{1g} \right) \right),$$

$$\frac{\partial Q}{\partial \beta_1} = \frac{\alpha_1 \sum_g \tilde{z}_{g1}}{\beta_1} - \left(\frac{n}{2} + \alpha_1 \right) \sum_g \frac{\tilde{z}_{g1}}{A_1 \left(\mu_1 - B_{1g} \right)^2 + C_{1g}}.$$

2. w.r.t. $\mu_2, k_2, \alpha_2, \beta_2$: Denote

$$\begin{aligned} A_2 =& \frac{n}{2(nk_2 + 1)}, \\ B_{2g} =& \frac{\sum_l d_{gl}}{n}, \\ C_{2g} =& \beta_2 + \frac{1}{2} \sum_l d_{gl}^2 - \frac{\left(\sum_l d_{gl} \right)^2}{2n}, \\ D_2 =& \alpha_2 \log \beta_2 + \log \Gamma \left(\frac{n}{2} + \alpha_2 \right) - \log \Gamma \left(\alpha_2 \right) - \frac{n}{2} \log(2\pi) - \frac{1}{2} \log(nk_2 + 1). \end{aligned}$$

Then we have

$$\frac{\partial Q}{\partial \mu_2} = - \left(\frac{n}{2} + \alpha_2 \right) \sum_g \left(\tilde{z}_{g2} \frac{2A_2 \left(\mu_2 - B_{2g} \right)}{A_2(\mu_2 - B_{2g})^2 + C_{2g}} \right),$$

$$\frac{\partial Q}{\partial k_2} = - \frac{n \sum_g \tilde{z}_{g2}}{2(nk_2 + 1)} + \frac{n^2}{2 \left(nk_2 + 1 \right)^2} \left(\frac{n}{2} + \alpha_2 \right) \sum_g \frac{\tilde{z}_{g2} (\mu_2 - B_{2g})^2}{A(\mu_2 - B_{2g})^2 + C_{2g}},$$

$$\frac{\partial Q}{\partial \alpha_2} = \sum_g \left(\log \beta_2 + \frac{\Gamma'(\frac{n}{2} + \alpha_2)}{\Gamma(\frac{n}{2} + \alpha_2)} - \frac{\Gamma'(\alpha_2)}{\Gamma(\alpha_2)} \right) - \sum_g \left(\tilde{z}_{g2} \log \left(A_2 \left(\mu_2 - B_{2g} \right)^2 + C_{2g} \right) \right),$$

$$\frac{\partial Q}{\partial \beta_2} = \frac{\alpha_2 \sum_g \tilde{z}_{g2}}{\beta_2} - \left(\frac{n}{2} + \alpha_2\right) \sum_g \frac{\tilde{z}_{g2}}{A_2 \left(\mu_2 - B_{2g}\right)^2 + C_{2g}}.$$

3. w.r.t. α_3, β_3. We have

$$\frac{\partial Q}{\partial \alpha_3} = \sum_g \left(\tilde{z}_{g3}\left(\log \beta_3 + \frac{\Gamma'(\frac{n}{2}+\alpha_3)}{\Gamma(\frac{n}{2}+\alpha_3)} - \frac{\Gamma'(\alpha_3)}{\Gamma(\alpha_3)}\right)\right) - \sum_g \left(\tilde{z}_{g3} \log\left(\beta_3 + \frac{1}{2}\sum_g d_{gl}^2\right)\right),$$

$$\frac{\partial Q}{\partial \beta_3} = \frac{\alpha_3 \sum_g \tilde{z}_{g3}}{\beta_3} - \left(\frac{n}{2} + \alpha_3\right) \sum_g \frac{\tilde{z}_{g3}}{\beta_3 + \frac{1}{2}\sum_g d_{gl}^2}.$$

We iterate the E-step and M-step until the difference of parameters $\boldsymbol{\pi}$ and $\boldsymbol{\psi}$ between two consecutive iterations is small or the number of iterations has reached the allowed maximum number.

D Initialization of the EM algorithm

To get initial estimates for the 10 parameters in $\boldsymbol{\psi}$ and the two parameters in $\boldsymbol{\pi}$, we first run *limma* to get raw p-value of moderated t-test for each gene probe, based on which we can partition the dataset into three clusters (OE, UE, and NE) and then estimate $\boldsymbol{\pi}$ by the cluster proportions. Next, within each cluster, we can use moment estimator to get crude estimates of the model parameters for each cluster (μ_1, k_1, α_1, and β_1 for OE; μ_2, k_2, α_2, and β_2 for UE; and α_3 and β_3 for NE). We used sample median and sample median absolute deviation (mad) to estimate mean and standard deviation. We then re-parameterize these estimated parameters to get a rough estimate of $\boldsymbol{\psi}$.

1. Initial estimates for $\mu_1, k_1, \alpha_1, \beta_1$:

$$\hat{\mu}_g^0 = \text{med}_l(d_{gl}), \hat{\tau}_g^0 = \left(\frac{1}{\text{mad}_l(d_{gl})}\right)^2.$$

$$\hat{\mu}_1^0 = \text{med}_g\left(\hat{\mu}_g^0\right), \hat{\alpha}_1^0 = \left(\frac{\text{med}_g(\hat{\tau}_g^0)}{\text{mad}_g(\hat{\tau}_g^0)}\right)^2, \hat{\beta}_1^0 = \frac{\text{med}_g(\hat{\tau}_g^0)}{\left(\text{mad}_g(\hat{\tau}_g^0)\right)^2}.$$

$$\hat{k}_1^0 = \left(\text{mad}_g(\hat{\mu}_g^0)\right)^2 \times \text{med}_g(\hat{\tau}_g^0).$$

2. Initial estimates for $\mu_2, k_2, \alpha_2, \beta_2$:

$$\hat{\mu}_g^0 = \text{med}_l(d_{gl}), \hat{\tau}_g^0 = \left(\frac{1}{\text{mad}_l(d_{gl})}\right)^2.$$

$$\hat{\mu}_2^0 = \text{med}_g\left(\hat{\mu}_g^0\right), \hat{\alpha}_2^0 = \left(\frac{\text{med}_g(\hat{\tau}_g^0)}{\text{mad}_g(\hat{\tau}_g^0)}\right)^2, \hat{\beta}_2^0 = \frac{\text{med}_g(\hat{\tau}_g^0)}{\left(\text{mad}_g(\hat{\tau}_g^0)\right)^2}.$$

$$\hat{k}_2^0 = \left(\text{mad}_g(\hat{\mu}_g^0)\right)^2 \times \text{med}_g(\hat{\tau}_g^0).$$

3. Initial estimates for α_3, β_3:

$$\hat{\tau}_g^0 = \left(\frac{1}{\text{mad}_l(d_{gl})}\right)^2,$$

$$\hat{\alpha}_3^0 = \left(\frac{\text{med}_g(\hat{\tau}_g^0)}{\text{mad}_g(\hat{\tau}_g^0)}\right)^2, \hat{\beta}_3^0 = \frac{\text{med}_g(\hat{\tau}_g^0)}{\left(\text{mad}_g(\hat{\tau}_g^0)\right)^2}.$$

E GEO data QC plots

We checked if there exist batch effects, outlying arrays, or outlying probes by drawing the trajectory plots of quantiles of log2 expression levels across arrays and the scatter plots of the first principal component and the second principal component. The three GEO data sets showed good quality. No outlying arrays or probes were detected. In PCA plot for GSE6631, cases and controls are separated in the directory of the second principal component. There are no obvious outliers or batch effects.

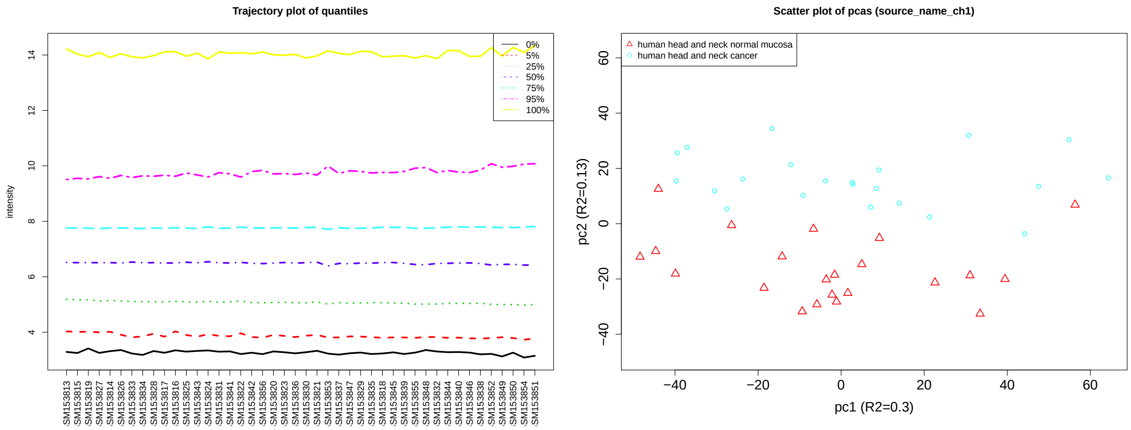

Figure A: QC plots for GSE6631. Left panel: plot of quantiles of log2 expression levels across arrays. Right panel: plot of the first principal component against the second principal component. Both plots show no obvious outliers or batch effects.

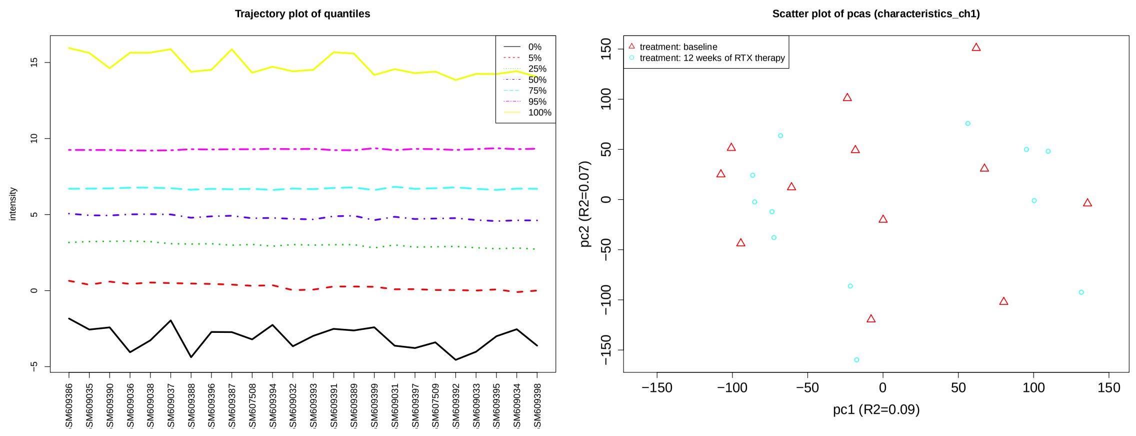

Figure B: QC plots for GSE24742. Left panel: plot of quantiles of log2 expression levels across arrays. Right panel: plot of the first principal component against the second principal component. Both plots show no obvious outliers or batch effects.

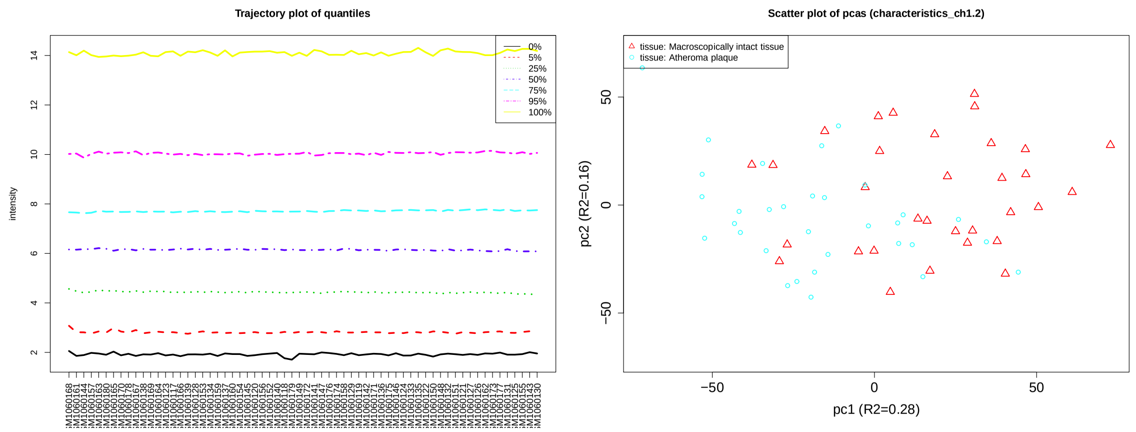

Figure C: QC plots for GSE43292. Left panel: plot of quantiles of log2 expression levels across arrays. Right panel: plot of the first principal component against the second principal component. Both plots show no obvious outliers or batch effects.

F Simulation results for scenarios with 100 pairs of samples

Here we present the simulation results for G100 and S100.

For all four simulations, we also summarized the results by using Figures D - I.

Table A: **Summary of agreement indices from simulation results for the scenario where $n = 100$ pairs per data set**

		eLNNpaired		limma		gt		samr		lmPerGene	
		mean	sd	mean	sd	mean	sd	mean	sd	mean	sd
Rand	G100	0.999	0.001	0.986	0.005	0.987	0.005	0.977	0.006	0.985	0.005
	S100	1.000	0.000	0.990	0.005	0.991	0.004	0.979	0.006	0.989	0.005
HA	G100	0.998	0.004	0.966	0.012	0.968	0.012	0.944	0.015	0.963	0.013
	S100	1.000	0.000	0.967	0.015	0.971	0.014	0.935	0.019	0.963	0.015
MA	G100	0.998	0.004	0.966	0.012	0.968	0.012	0.944	0.015	0.963	0.013
	S100	1.000	0.000	0.967	0.015	0.971	0.014	0.935	0.019	0.963	0.015
FM	G100	0.999	0.001	0.990	0.004	0.991	0.004	0.984	0.004	0.989	0.004
	S100	1.000	0.000	0.994	0.003	0.994	0.003	0.987	0.004	0.993	0.003
Jaccard	G100	0.999	0.002	0.981	0.007	0.982	0.007	0.968	0.008	0.979	0.008
	S100	1.000	0.000	0.987	0.006	0.989	0.005	0.975	0.008	0.986	0.006

Table B: **Summary of error rates from simulation results for the scenario where $n = 100$ pairs per data set**

		eLNNpaired		limma		gt		samr		lmPerGene	
		mean	sd	mean	sd	mean	sd	mean	sd	mean	sd
FDR	G100	0.000	0.001	0.043	0.015	0.042	0.015	0.073	0.018	0.049	0.016
	S100	0.000	0.000	0.050	0.022	0.045	0.021	0.097	0.027	0.056	0.023
FNDR	G100	0.001	0.001	0.000	0.001	0.000	0.001	0.000	0.000	0.000	0.001
	S100	0.000	0.000	0.000	0.000	0.000	0.000	0.000	0.000	0.000	0.000
FPR	G100	0.000	0.000	0.009	0.003	0.008	0.003	0.015	0.004	0.010	0.003
	S100	0.000	0.000	0.006	0.003	0.005	0.003	0.012	0.004	0.007	0.003
FNR	G100	0.003	0.005	0.002	0.003	0.002	0.004	0.001	0.002	0.002	0.003
	S100	0.000	0.000	0.000	0.000	0.000	0.000	0.000	0.000	0.000	0.000

G Comparing the power of the significant probes to predict disease status

For each of the three GEO data sets, we applied random forest algorithm to build prediction models based on the differentially expressed (DE) gene probes obtained by *eLNNpaired*,

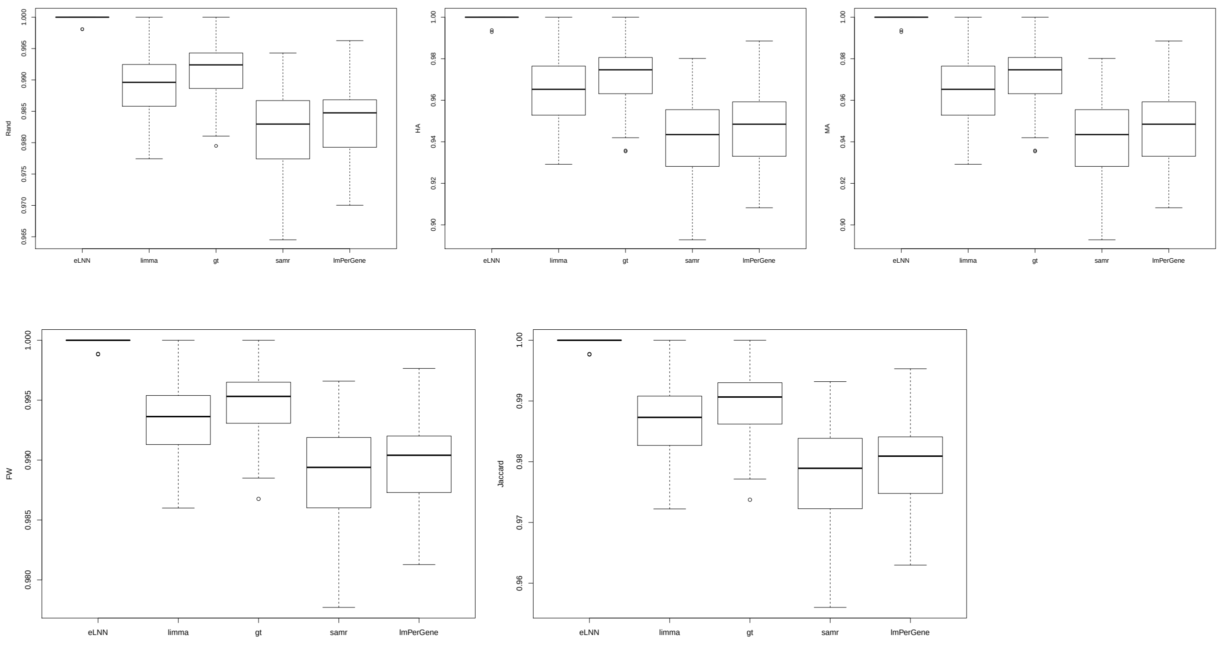

Figure D: Boxplots of the agreement indices (Rand, HA, MA, FM, and Jaccard) for *eLNNpaired*, *limma*, *gt*, *samr*, and *lmPerGene* based on simulation S30. Top-left panel: Rand; Top-middle panel: HA; Top-right panel: MA; Bottom-left panel: FM; Bottom-right panel: Jaccard.

limma, *gt*, *samr*, and *lmPerGene*, respectively. We then predicted disease statuses of subjects and drew ROC curves (Figure J) and precision-recall curves (Figure K).

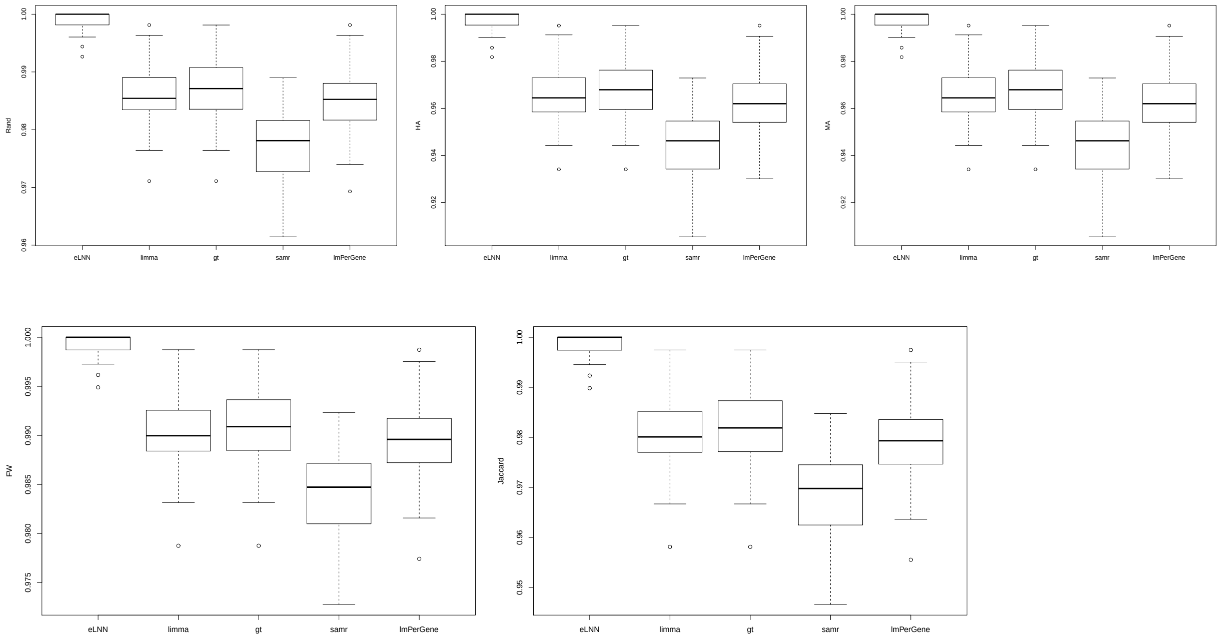

Figure E: Boxplots of the agreement indices (Rand, HA, MA, FM, and Jaccard) for *eLNNpaired*, *limma*, *gt*, *samr*, and *lmPerGene* based on simulation G100. Top-left panel: Rand; Top-middle panel: HA; Top-right panel: MA; Bottom-left panel: FM; Bottom-right panel: Jaccard.

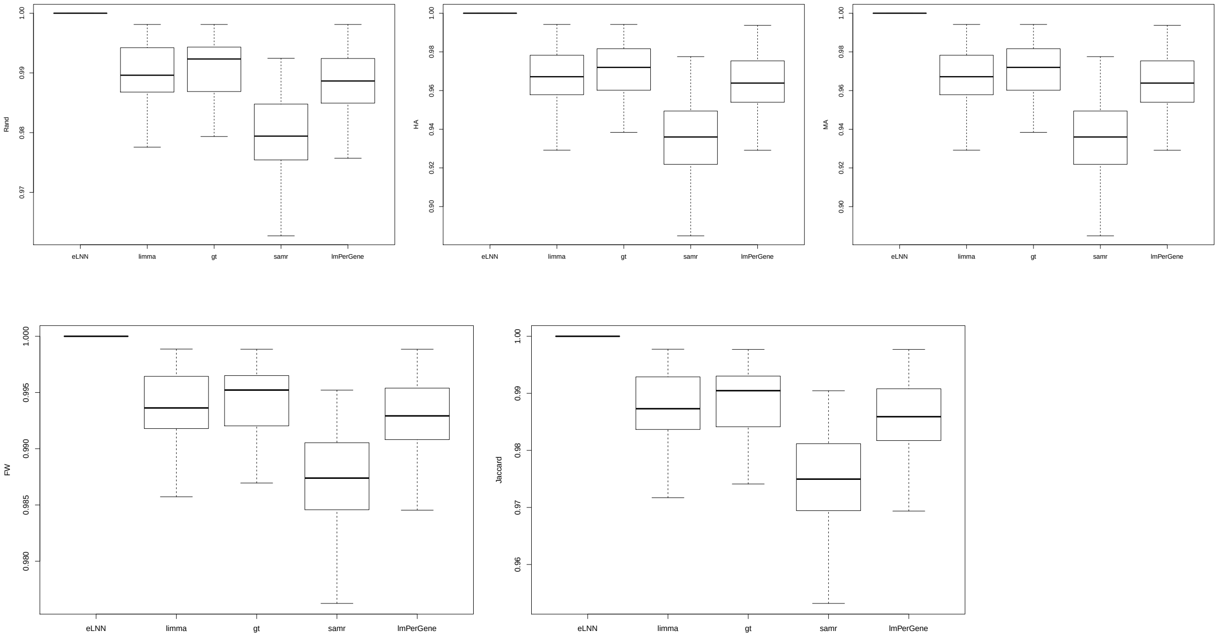

Figure F: Boxplots of the agreement indices (Rand, HA, MA, FM, and Jaccard) for *eLNNpaired*, *limma*, *gt*, *samr*, and *lmPerGene* based on simulation S100. Top-left panel: Rand; Top-middle panel: HA; Top-right panel: MA; Bottom-left panel: FM; Bottom-right panel: Jaccard.

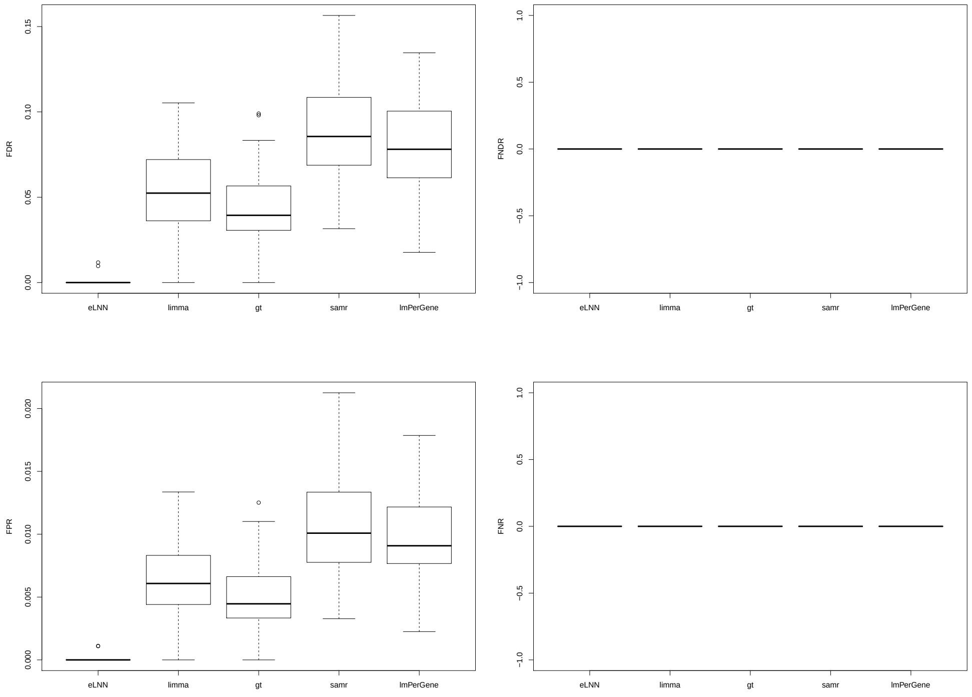

Figure G: Boxplots of the error rates (FDR, FNDR, FNR, and FPR) for *eLNNpaired*, *limma*, *gt*, *samr*, and *lmPerGene* based on simulation S30. Top-left panel: FDR; Top-right panel: FNDR; Bottom-left panel: FPR; Bottom-right panel: FNR.

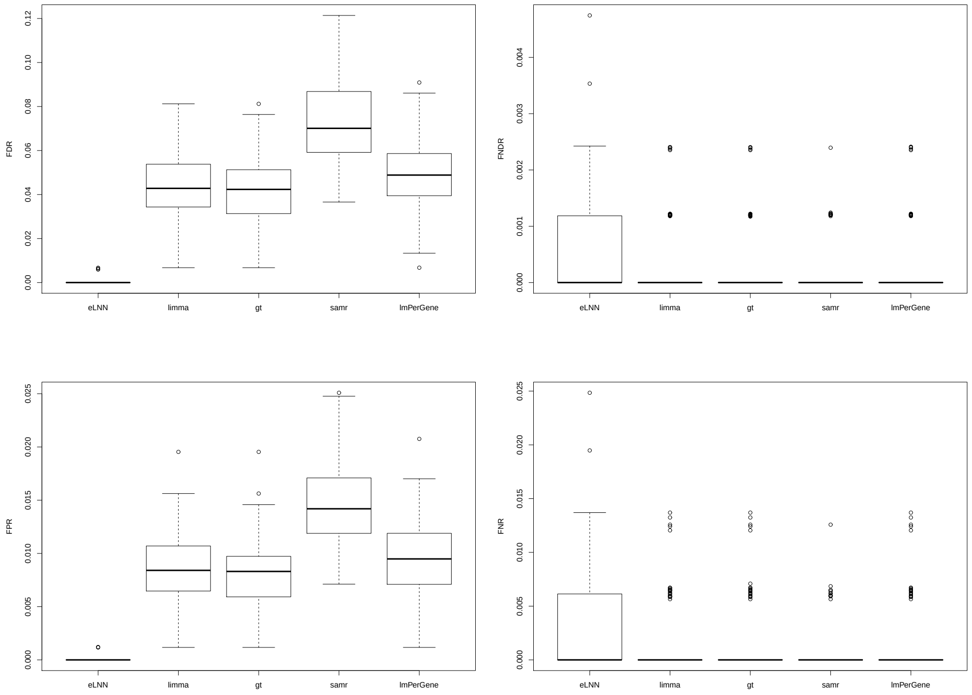

Figure H: Boxplots of the error rates (FDR, FNDR, FNR, and FPR) for *eLNNpaired*, *limma*, *gt*, *samr*, and *lmPerGene* based on simulation G100. Top-left panel: FDR; Top-right panel: FNDR; Bottom-left panel: FPR; Bottom-right panel: FNR.

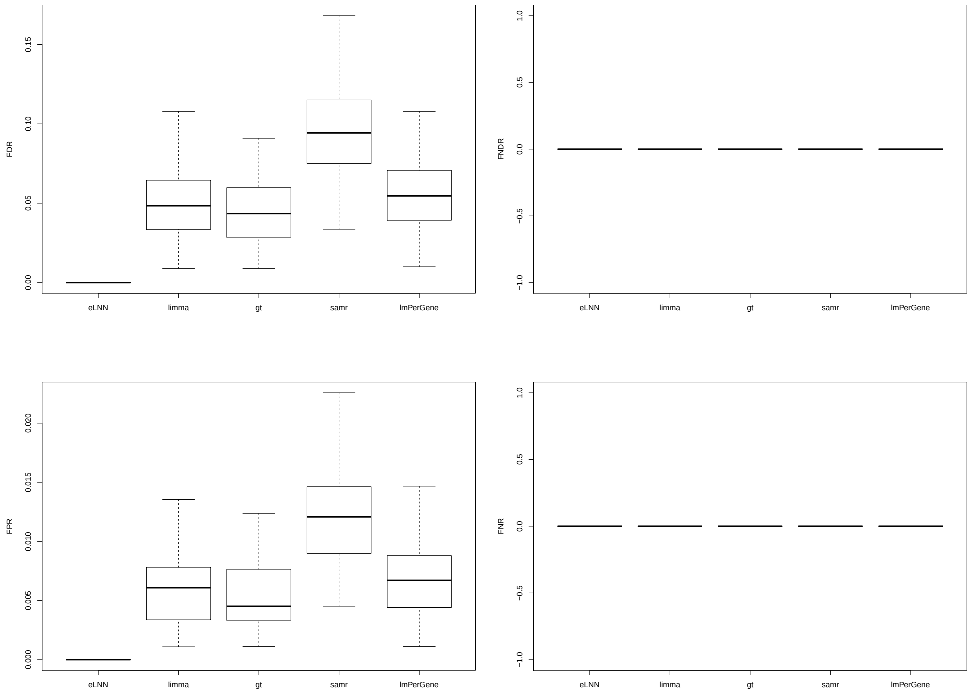

Figure I: Boxplots of the error rates (FDR, FNDR, FNR, and FPR) for *eLNNpaired*, *limma*, *gt*, *samr*, and *lmPerGene* based on simulation S100. Top-left panel: FDR; Top-right panel: FNDR; Bottom-left panel: FPR; Bottom-right panel: FNR.

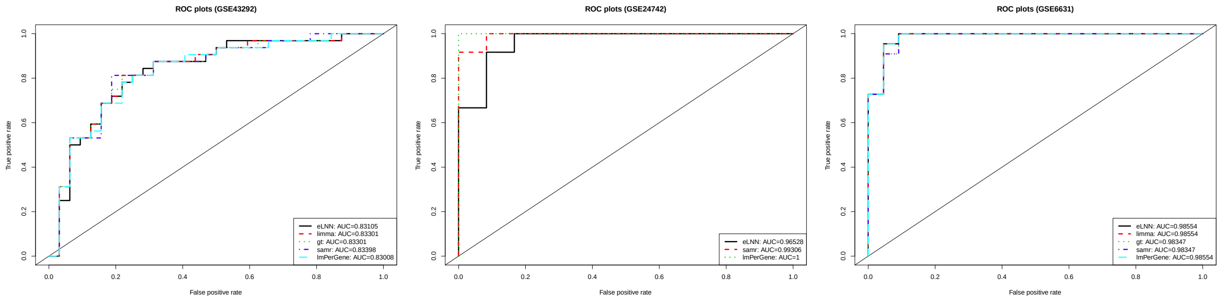

Figure J: ROC curves for the 3 GEO data sets. Left panel: GSE43292; middle panel: GSE24742; right panel: GSE6631. Black solid line indicates *eLNNpaired*. The closer a ROC toward to upper left corner, the more prediction power. AUC stands for area under ROC. The closer an AUC to one, the more prediction power.

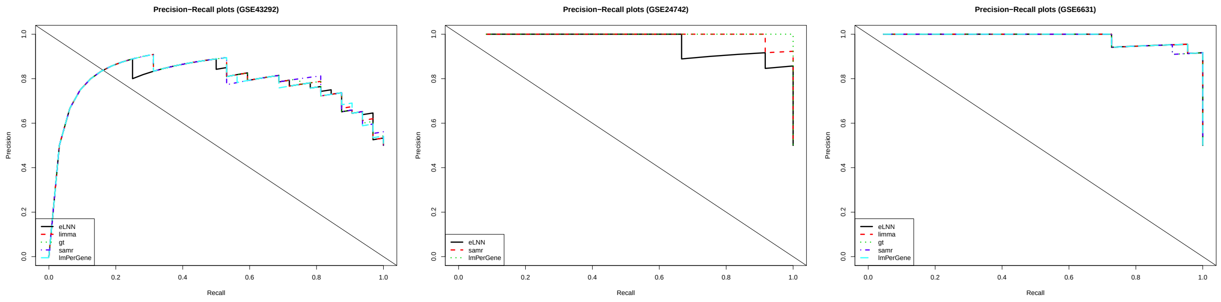

Figure K: Precision-recall curves for the 3 GEO data sets. Left panel: GSE43292; middle panel: GSE24742; right panel: GSE6631. Black solid line indicates *eLNNpaired*. The closer a precision-recall curve toward to upper right corner, the more prediction power.

References

[1] Kendziorski, CM, Newton, MA, Lan, H, and Gould, MN27. On parametric empirical bayes methods for comparing multiple groups using replicated gene expression profiles. *Statistics in medicine*, 22(24):3899–3914, 2003.

[2] Lo, K and Gottardo, R. Flexible empirical bayes models for differential gene expression. *Bioinformatics*, 23:328–335, 2007.

[3] Newton, MA, Kendziorski, CM, Richmond, CS, Blattner, FR, and Tsui, KW. On differential variability of expression ratios: improving statistical inference about gene expression changes from microarray data. *Journal of computational biology*, 8(1):37–52, 2001.
